# Integrative morpho-molecular delineation of five medically significant tick species: facilitating precision-based vector surveillance

**DOI:** 10.3389/fvets.2025.1623318

**Published:** 2025-08-08

**Authors:** Guangxin Shi, Lianxi Xin, Zhuocheng Li, Wanpeng Ma, Depeng Yang, Caishan Li, Bayin Chahan, Qingyong Guo

**Affiliations:** ^1^Parasitology Laboratory, Veterinary College, Xinjiang Agricultural University, Ürümqi, China; ^2^Muwang Veterinary Service Co., Ltd, Jimusaer, China

**Keywords:** tick, *Hyalomma anatolicum*, *Hyalomma asiaticum*, *Hyalomma dromedarii*, *Rhipicephalus turanicus*, *Dermacentor marginatus*, Identification

## Abstract

Ticks serve as major vectors of zoonotic pathogens, posing significant threats to public health and livestock. However, morphological similarity among closely related species complicates accurate identification. This study employed an integrative taxonomic approach combining morphological and molecular methods to delineate five medically important tick species in Xinjiang, China. From April to July 2024, a total of 1,128 ticks were collected from livestock across four ecological regions (Ürümqi, Turpan, Jimsar, and Aksu). Morphological features—including basis capituli, scutum, and genital aperture—were analyzed via stereomicroscopy, while molecular characterization targeted mitochondrial (16S rDNA, COI) and nuclear (ITS2) markers. Phylogenetic trees were reconstructed using the maximum likelihood method. Morphological identification confirmed five species: *Hyalomma anatolicum, Hyalomma asiaticum, Hyalomma dromedarii, Rhipicephalus turanicus*, and *Dermacentor marginatus*, supported by high-resolution imaging. Molecular data revealed notable interregional genetic affinities: *H. anatolicum* from Turpan shared COI similarity with strains from Kazakhstan; *H. asiaticum* from Turpan clustered with Iranian populations; *H. dromedarii* from Jimsar showed 16S rDNA similarity to Saudi Arabian lineages; *R. turanicus* from Aksu grouped with Egyptian COI sequences; and *D. marginatus* from Jimsar aligned with populations from Alashankou, China. Notably, single-gene phylogenies posed taxonomic limitations—for example, ITS2 misclassified *R. turanicus* as *Rhipicephalus sanguineus sensu stricto*. These issues were mitigated through morphological diagnostics such as scutal enamel spots in *D. marginatus* and the distinctive genital plates in *H. dromedarii*. The integrative approach improved taxonomic resolution, with 16S rDNA providing genus-level insight, COI enabling species-level discrimination, and ITS2 offering strain-level resolution. Additionally, high-resolution morphological imaging addressed gaps in existing reference databases. Overall, multi-locus strategies combined with morphological validation are essential for accurate tick identification, improving our capacity to monitor vector-borne pathogen transmission and contributing to One Health surveillance frameworks.

## Graphical Abstract

**Figure:**
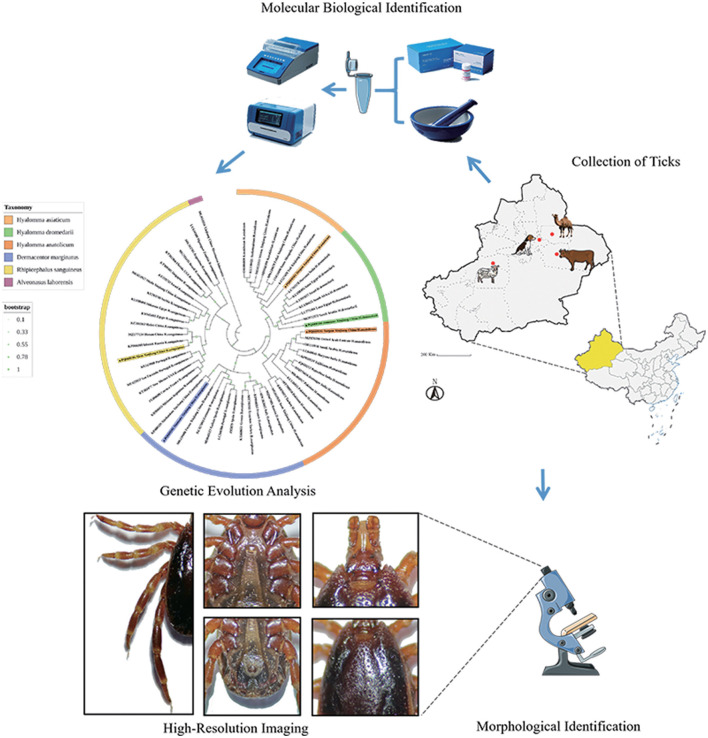

## 1 Introduction

Ticks are globally distributed, with ~896 species identified to date. In China alone, over 110 species of hard ticks (Ixodidae) have been recorded (1–4). Xinjiang, the largest province in China by land area, encompasses diverse geographical and environmental conditions that favor the proliferation of multiple tick species (5, 6). As obligate *hematophagous ectoparasites*, ticks represent the second most significant group of biological vectors after mosquitoes, requiring blood meals at every developmental stage (1, 2, 7). Their feeding behavior causes tissue damage through skin penetration and facilitates the transmission of a wide range of tick-borne pathogens (8–10). Notably, some pathogens can be vertically transmitted via tick eggs (7), thereby expanding their spatial and temporal transmission potential. These attributes collectively pose substantial threats to public health and the livestock industry (8, 11, 12). Different tick species are associated with distinct pathogen profiles—for instance, *Hyalomma anatolicum* primarily transmits *Theileria annulata*, whereas its congener *Hyalomma asiaticum* is a major vector of Crimean-Congo Hemorrhagic Fever in Central Asia and China. Accurate tick species identification is therefore crucial from both medical and veterinary perspectives, enabling early intervention and containment of emerging tick-borne diseases.

Morphological identification of tick species relies on key structural features, including the shape of the basis capituli, the sulci and ornamentation of the dorsal scutum, leg spur configurations, and ventral plate morphology. However, this process requires significant taxonomic expertise due to the subtle and often overlapping morphological traits among species (13–15). Molecular identification methods, primarily based on polymerase chain reaction (PCR) amplification of targeted genetic regions, have become increasingly prevalent. Commonly employed genetic markers for tick identification and phylogenetic analysis include the 16S ribosomal RNA gene (16S rDNA), cytochrome c oxidase subunit I (COI), and the internal transcribed spacer 2 (ITS2). The 16S rDNA comprises 10 conserved and nine variable regions; the former display high sequence homology across taxa, reflecting deep phylogenetic relationships, while the latter exhibit species-specific variations that align with evolutionary divergence patterns (16, 17). COI, a key component of the mitochondrial respiratory chain, is widely used as a molecular barcode for accurate tick species classification and is often referred to as the “DNA barcode” for ticks (18, 19). ITS2, a non-coding nuclear region within the eukaryotic ribosomal RNA gene cluster, incorporates biparental genetic contributions and offers robust phylogenetic resolution due to its high interspecific variability (20, 21).

Northern Xinjiang's Urumqi region represents a hotspot for tick-borne disease diversity, predominantly involving *Hyalomma* and *Rhipicephalus* species, and is associated with Lyme disease, tick-borne encephalitis, and emerging bunyaviral infections (22). In contrast, Turpan, dominated by *Hyalomma* ticks, is a key region for investigating livestock-associated pathogens such as bovine theileriosis, Crimean-Congo hemorrhagic fever, and Q fever (6, 23). Jimsar, influenced by climate change-driven northward tick expansion, has become a pivotal transitional zone for monitoring the genotypic evolution of *Borrelia burgdorferi* and the cross-border transmission of tick-borne relapsing fever (24). Aksu, situated near Central Asian epidemic zones, serves as a strategic frontier for tracking transboundary pathogen incursions and studying tick–symbiont coevolution dynamics (25). Together, these regions span Xinjiang's major ecological gradients—forest-steppe, extreme arid zones, transitional belts, and cross-border interfaces—providing an ideal framework for systematically investigating tick-borne pathogen diversity, transmission dynamics, and control strategies. Enhancing the precision of tick identification in these regions is thus of paramount importance.

A major challenge in current tick taxonomy stems from limited access to high-resolution morphological imaging and the presence of intraspecific morphological variability, which complicates identification and has driven a growing dependence on molecular methods. However, this reliance has also led to taxonomic ambiguities, particularly among closely related congeneric species. To address these issues, this study integrates traditional morphological identification with molecular phylogenetic analysis of ticks collected from the four aforementioned regions. High-resolution morphological imaging is combined with mitochondrial (e.g., *cox1*) and nuclear (e.g., *ITS2*) genetic markers to resolve evolutionary relationships and minimize misclassification. Moreover, this integrative approach facilitates elucidation of the genetic relatedness of ticks across the Central Asia–Middle East region and addresses existing gaps in diagnostic imaging datasets.

## 2 Materials and methods

### 2.1 Sample collection

**Figure 1:**
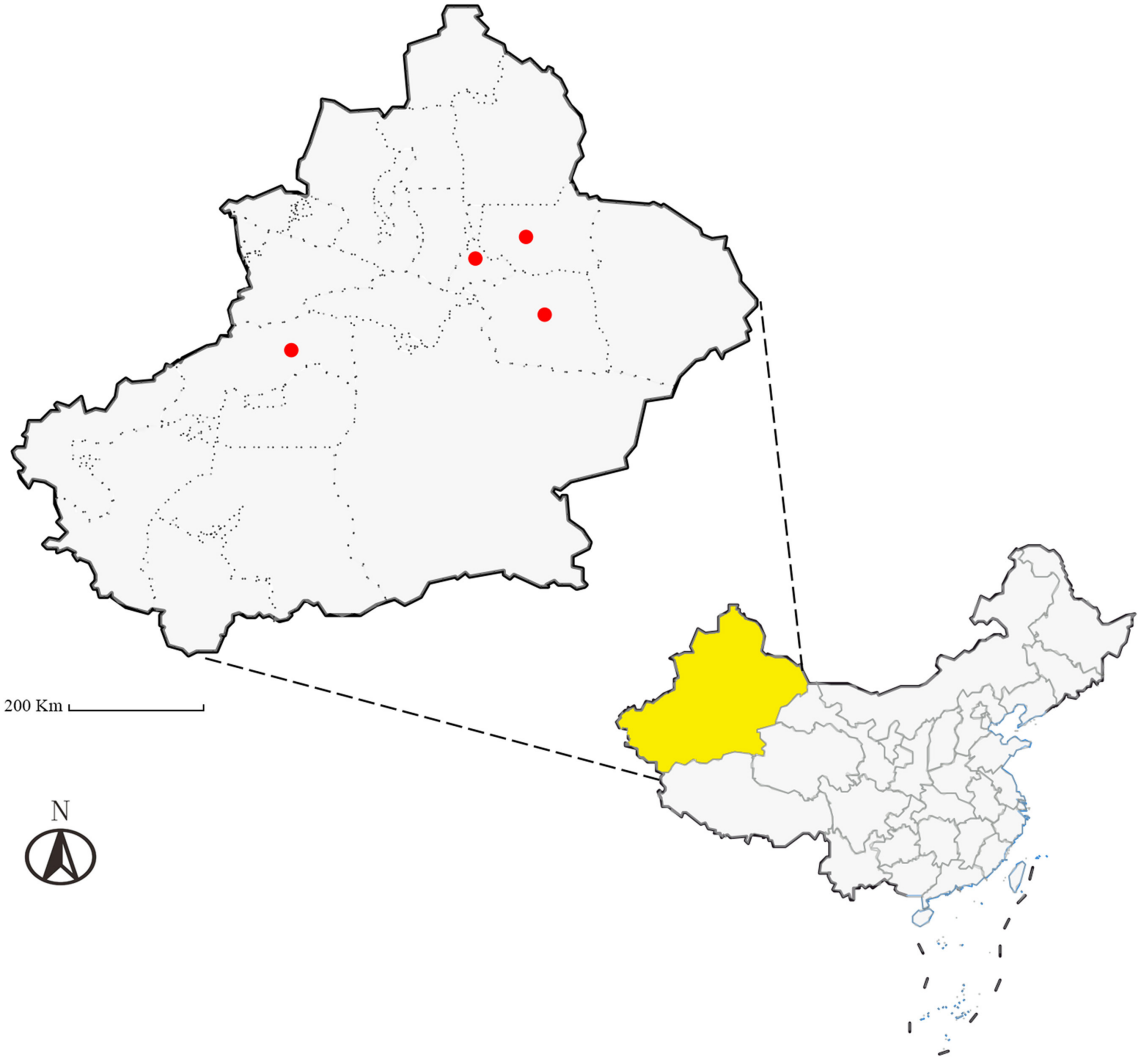
Schematic map of the study sampling sites (Ürümqi City, Turpan City, Jimsar County, and Aksu Prefecture in Xinjiang, China), Scale bar = 200 Km.

Between April and July 2024, a total of 1,128 ticks were collected across Xinjiang (see Table 1; distribution shown in Figure 1) from hosts including *Simmental cattle, Altay sheep, Bactrian camel*s, and *Chinese rural* dogs. Ticks were housed in perforated containers sealed with cotton gauze and transported to the Parasitology Laboratory, College of Veterinary Medicine, Xinjiang Agricultural University, for preservation and analysis.

**Table 1:** Data on the hosts and geographical origin of ticks (*n* = 1,128) included in this study.

**Species**	**Geographical origin**	**Geographical coordinates**	**Climate and vegetation**	**Hosts**
*H. anatolicum*	Turpan (453)	89°11′04″E, 42°55′50″N	Extreme arid desert (Halophytic meadows)	*Simmental cattle, Altay sheep*
*H. asiaticum*	Turpan (335)	89°15′47″E, 42°42′02″N	Extreme arid desert (Oasis crops)	*Simmental cattle, Altay sheep*
*H. dromedarii*	Jimsar (50)	88°53′39″E, 44°04′08″N	Temperate continental (Desert *Tamarix*)	*Bactrian camel*
*R. turanicus*	Aksu (141)	80°23′16″E, 40°38′58″N	Continental arid (River valley *Populus euphratica*)	*Altay sheep*
	Urumqi (53)	87°35′08″E, 43°51′10″N	Mid-temperate arid continental (Plantation forests)	*Chinese rural dog*
*D. marginatus*	Jimsar (96)	89°11′14″E, 44°00′31″N	Temperate continental (Plain crops)	*Altay sheep*

### 2.2 Morphological identification and high-resolution imaging

Specimens were alternately rinsed with 75% ethanol and physiological saline to eliminate surface contaminants. Morphological examination was conducted using an SMZ161 stereomicroscope, following standardized protocols from relevant literature [references (26–29) and Books (13–15)]. Key morphological features—including the capitulum, scutum, spiracular plates, anal plates, legs, and pulvilli—were assessed for preliminary taxonomic classification. Specimens were preserved in 75% ethanol and subsequently subjected to molecular validation. High-resolution imaging was performed post-genetic confirmation.

### 2.3 Genomic DNA extraction

In each sampling area, three live ticks per species exhibiting consistent morphological characteristics were selected. Specimens were flash-frozen in liquid nitrogen and homogenized. Genomic DNA was extracted under low-temperature conditions using a commercial kit (Tiangen Biochemical Technology Co., Ltd.), according to the manufacturer's protocol. Extracted DNA was stored at −20°C for downstream applications.

### 2.4 PCR amplification of the target gene

PCR amplification targeted three genetic loci using the following primer sets: 16S rDNA: Forward (F): 5′-TTAAATTGCTGTRGTATT-3′; Reverse (R): 5′-CCGGTCTGAACTCASAWC-3′. Tick COI gene: Forward (F): 5′-GGTCAACAAATCATAAAGATATTGG-3′; Reverse (R): 5′-TAAACTTCAGGGTGACCAAAAAATCA-3′. Tick ITS2 gene: Forward (F): 5′-TCGTCTGTCTGAGGGTCGGA-3′; Reverse (R): 5′-ATCGTCTCGTGTAGCGTCG-3′.

Each 25-μL PCR reaction system included 12.5 μl of 2 × Taq PCR Master Mix, 1.0 μl each of forward and reverse primers (10 μmol/L), 1.0 μl DNA template, and 9.5 μl ddH_2_O. Thermal cycling conditions for 16S rDNA gene amplification were as follows: 94°C initial denaturation for 3 min; 35 cycles of 94°C denaturation for 30 s, 43.6°C annealing for 30 s, and 72°C extension for 1 min; followed by a final extension step at 72°C for 10 min. For the COI gene, the conditions were as follows: 94°C initial denaturation for 3 min; 35 cycles of 94°C denaturation for 30 s, 51.5°C annealing for 30 s, and 72°C extension for 1 min; with a final extension step at 72°C for 10 min. The ITS2 gene amplification protocol was as follows: 94°C initial denaturation for 3 min; 35 cycles of 94°C denaturation for 30 s, 57.2°C annealing for 30 s, and 72°C extension for 1 min; concluding with a final extension at 72°C for 10 min.

Negative controls using genomic DNA from *Mus musculus* (Kunming mouse strain) blood samples were included in all PCR assays. Amplicons were separated by 1.5% agarose gel electrophoresis. Target DNA fragments were extracted, purified using a standard agarose gel DNA extraction kit (Tiangen Biochemical Technology Co., Ltd.) following the manufacturer's protocol and subsequently subjected to Sanger sequencing (Sangon Biotech Co., Ltd.).

### 2.5 Sequence alignment and phylogenetic analysis

Raw sequences were aligned against the National Center for Biotechnology Information (NCBI) database to identify homologous tick sequences. Primer sequences were trimmed, and curated sequences were uploaded to the NCBI repository. Reference sequences corresponding to conspecific ticks were retrieved, while those with consecutive accession numbers, incomplete metadata (e.g., uncertain collection localities), or ambiguous nucleotide content or length were excluded.

Multiple sequence alignments were performed using the ClustalW algorithm in MEGA 11. Optimal nucleotide substitution models were determined as follows: Kimura 2-parameter with gamma distribution and invariant sites (G+I) for 16S rDNA; Tamura-Nei with G+I for COI; and Tamura 3-parameter with gamma distribution (G) for ITS2. Phylogenetic trees were constructed using the Maximum Likelihood method. *Alveonasus lahorensis* (for 16S rDNA and COI) and *Argas persicus* (for ITS2) were designated as outgroups for phylogenetic inference.

## 3 Results

### 3.1 Morphological identification results

**Figure 2:**
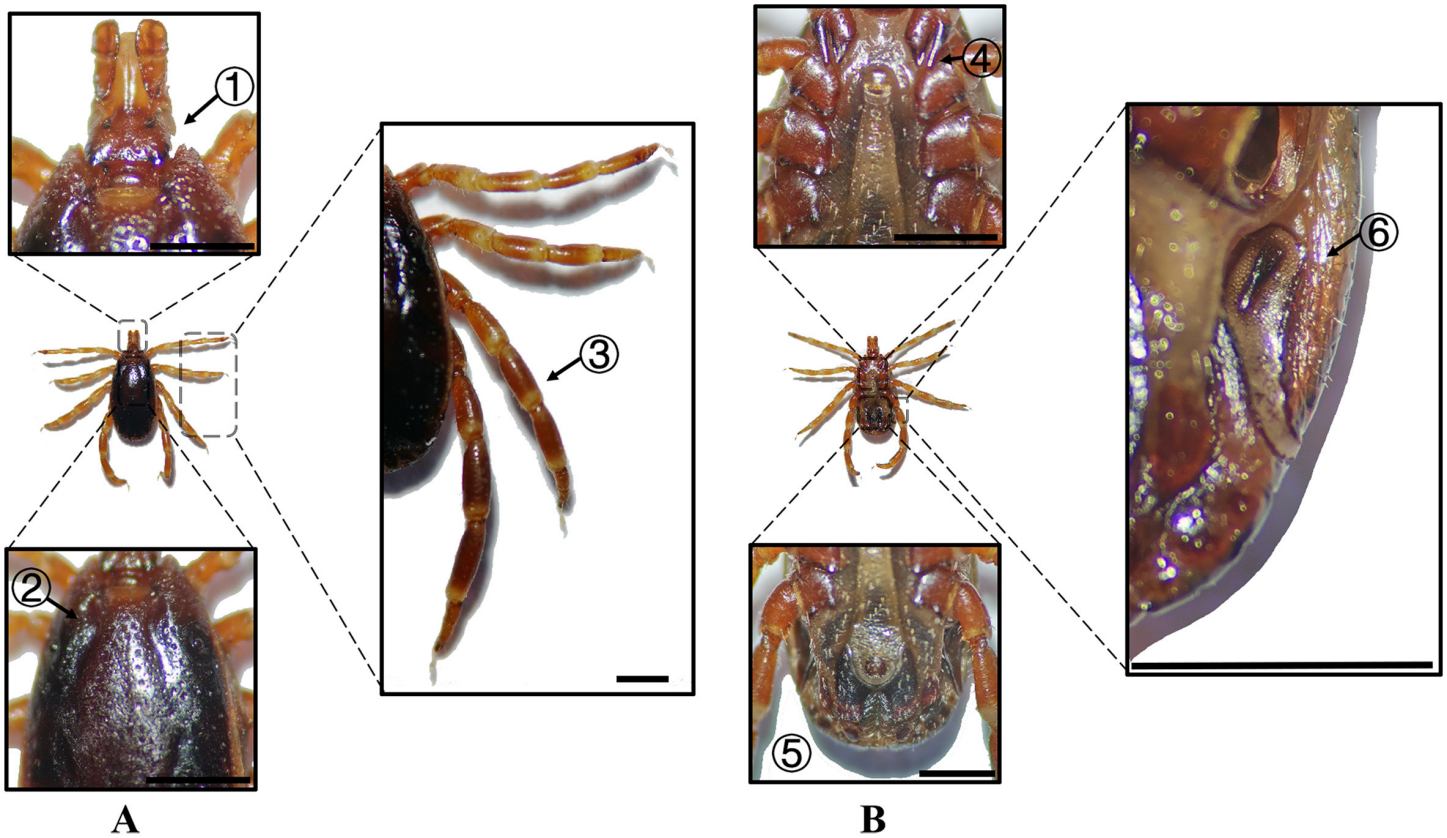
**(A)** Dorsal and **(B)** ventral views of a male *H. anatolicum*. Labels: (1) Basis capituli; (2) Cervical and lateral grooves; (3) Leg articulations; (4) External and internal spurs of coxae I; (5) Anal sulcus; (6) Spiracular plate. Scale bar = 1 mm.

**Figure 3:**
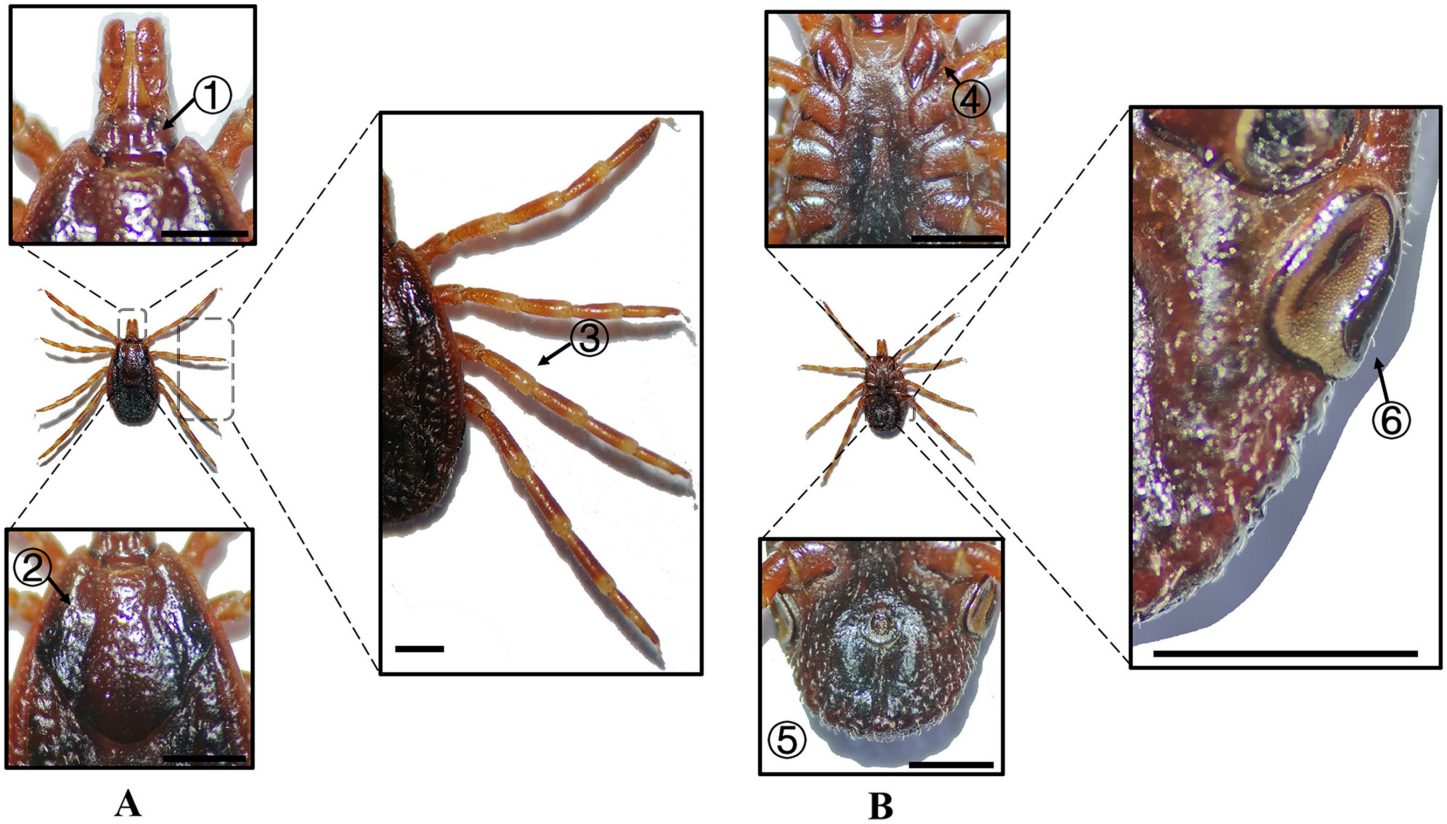
**(A)** Dorsal and **(B)** ventral views of a female *H. anatolicum*. Labels: (1) Basis capituli; (2) Cervical and lateral grooves; (3) Leg articulations; (4) External and internal spurs of coxae I; (5) Anal sulcus; (6) Spiracular plate. Scale bar = 1 mm.

**Figure 4:**
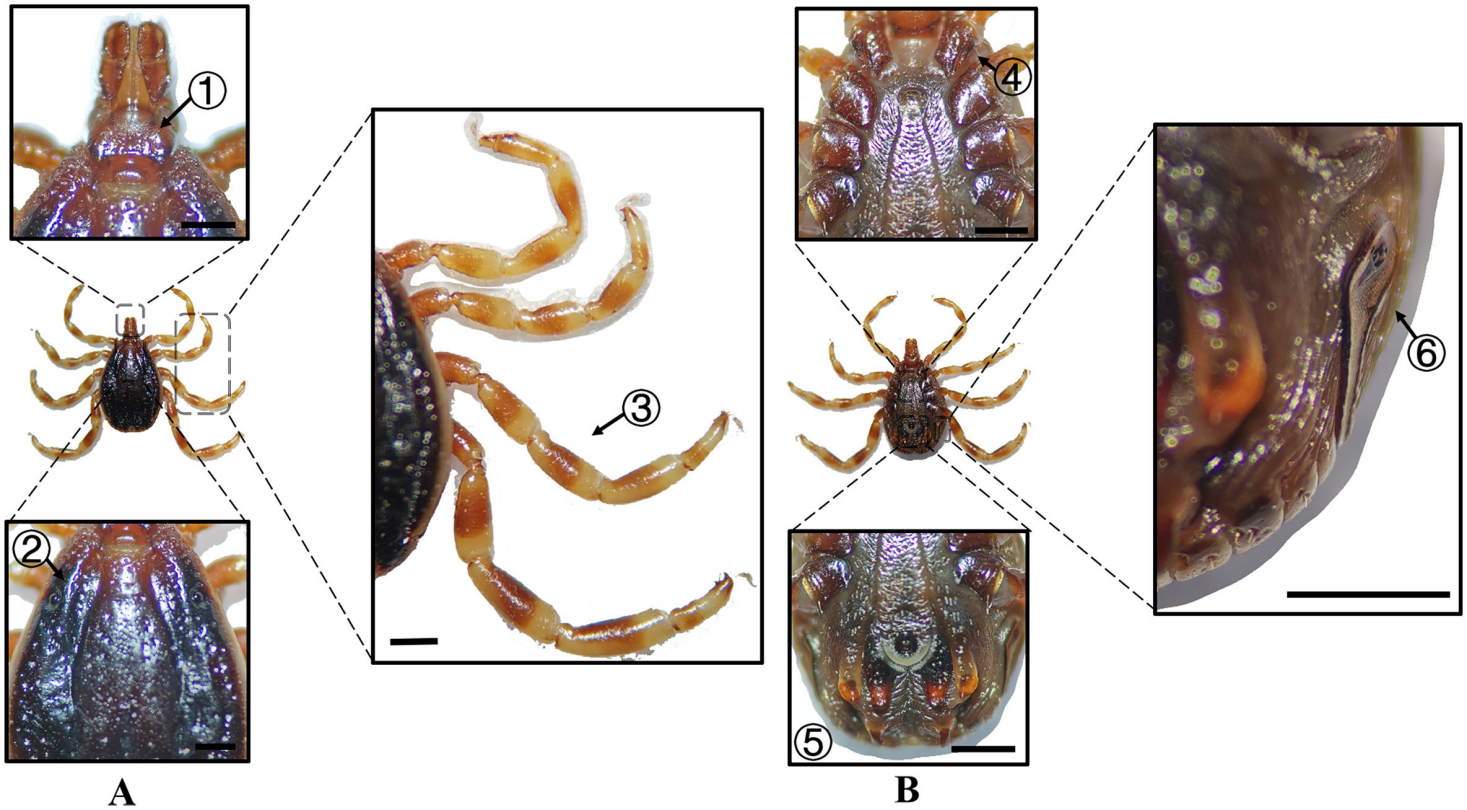
**(A)** Dorsal and **(B)** ventral views of a male *H. asiaticum*. Labels: (1) Basis capituli; (2) Cervical and lateral grooves; (3) Leg articulations; (4) External and internal spurs of coxae I; (5) Anal sulcus; (6) Spiracular plate. Scale bar = 1 mm.

**Figure 5:**
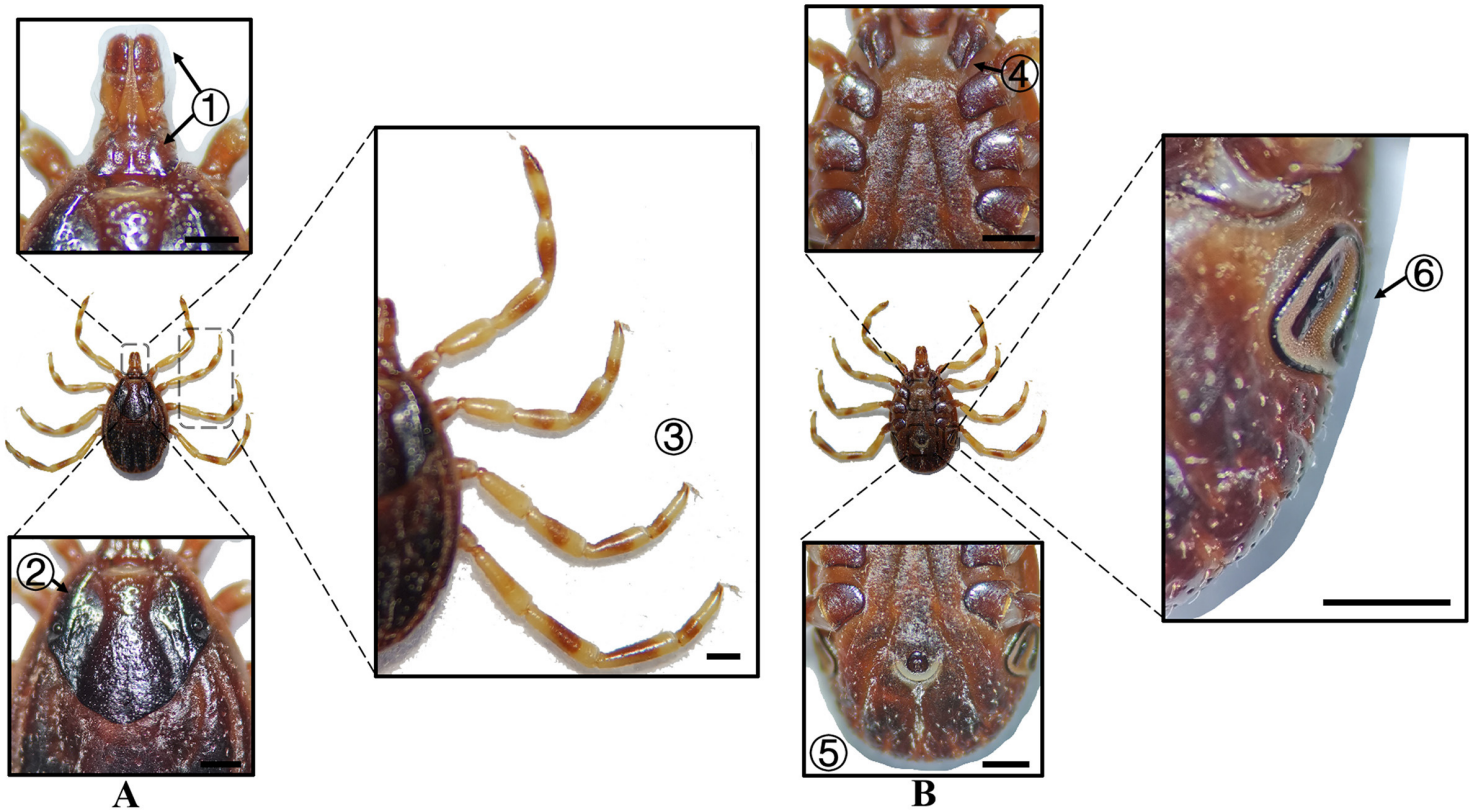
**(A)** Dorsal and **(B)** ventral views of a female *H. asiaticum*. Labels: (1) Basis capituli; (2) Cervical and lateral grooves; (3) Legs; (4) External and internal spurs of coxae I; (5) Anal sulcus; (6) Spiracular plate. Scale bar = 1 mm.

**Figure 6:**
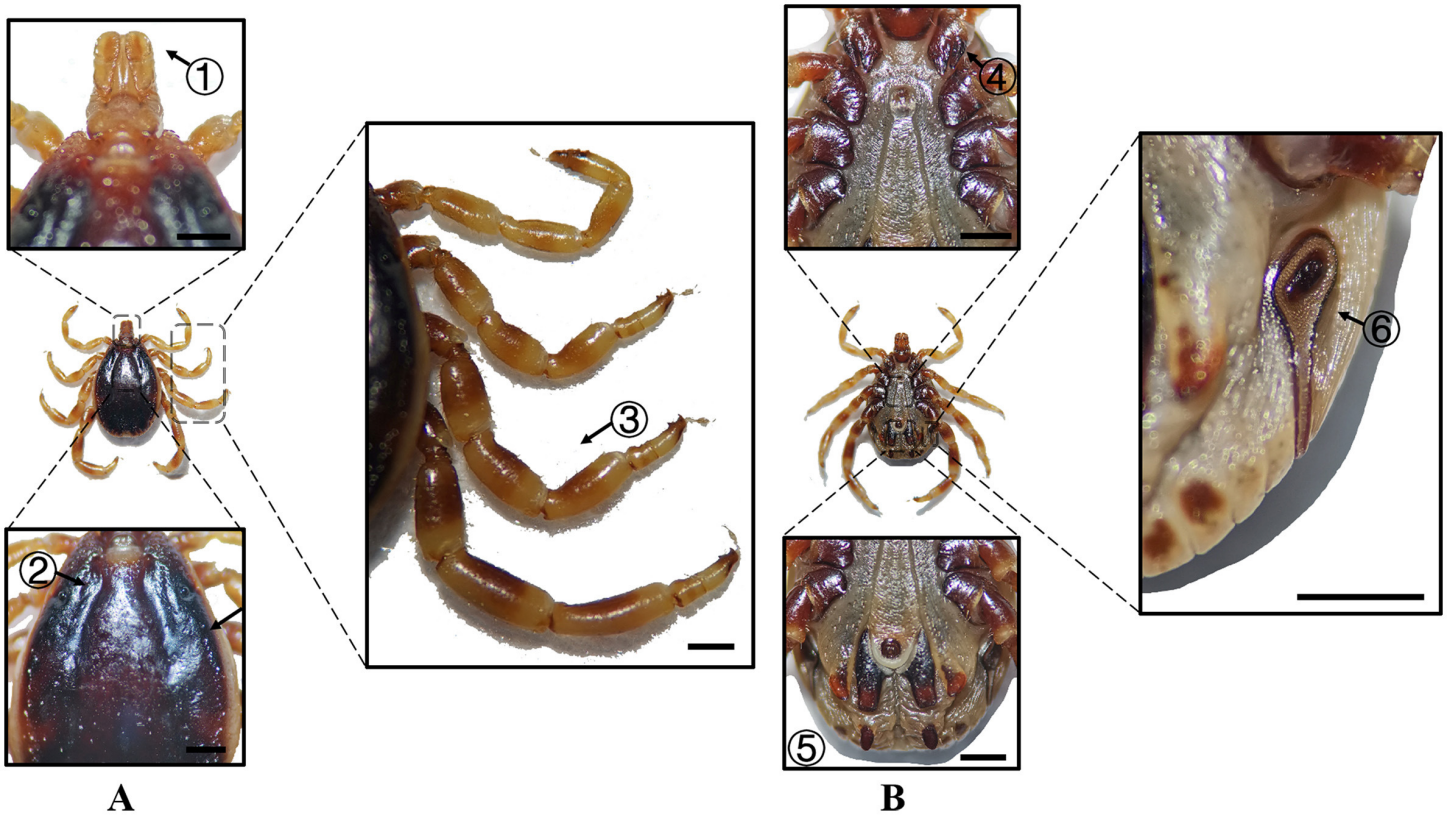
**(A)** Dorsal and **(B)** ventral views of a male *H. dromedarii*. Labels: (1) Palpi; (2) Cervical and lateral grooves; (3) Legs; (4) External and internal spurs of coxae I; (5) Anal sulcus; (6) Spiracular plate. Scale bar = 1 mm.

**Figure 7:**
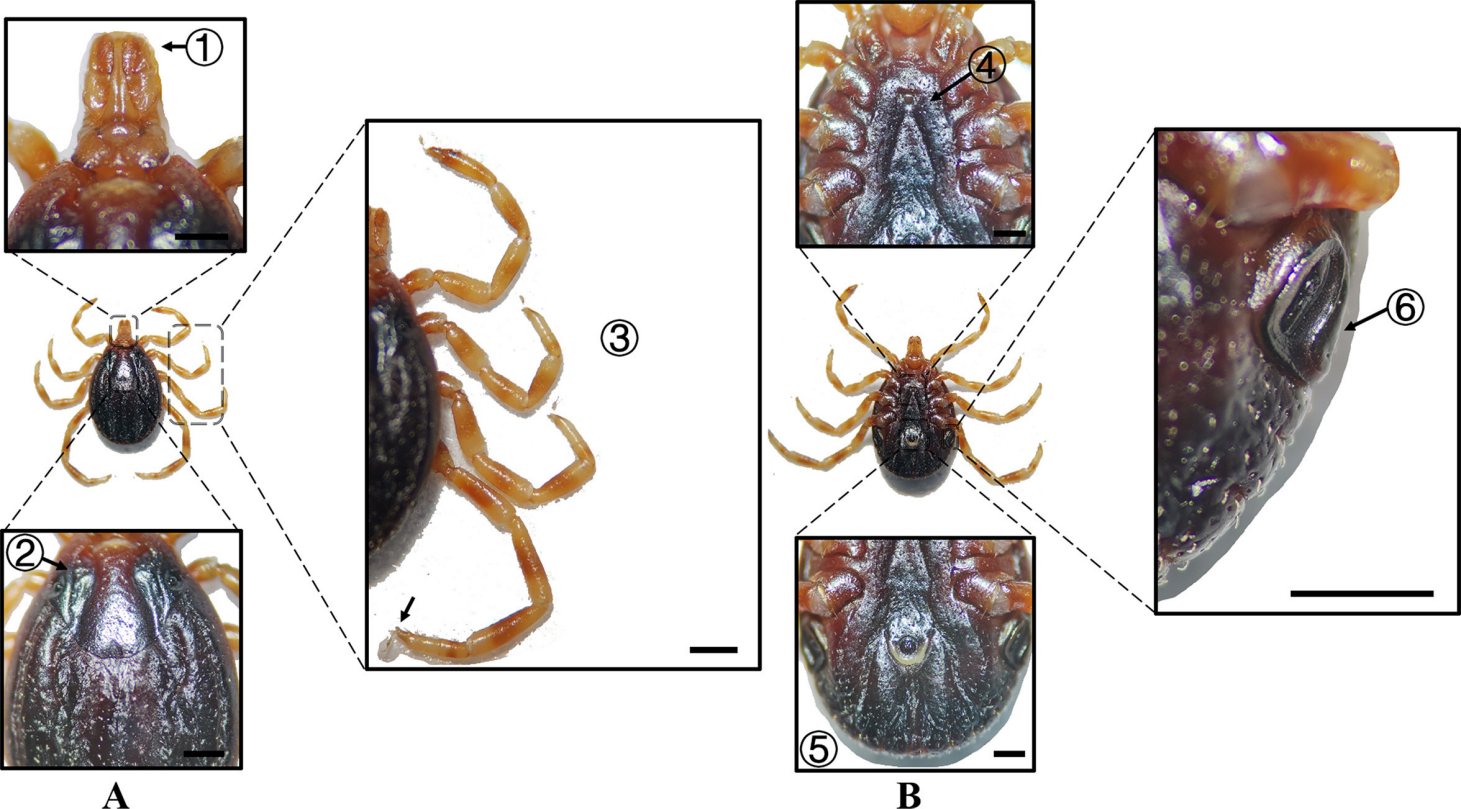
**(A)** Dorsal and **(B)** ventral views of a female *H. dromedarii*. Labels: (1) Palpi; (2) Cervical and lateral grooves; (3) Legs; (4) External and internal spurs of coxae I; (5) Anal sulcus; (6) Spiracular plate. Scale bar = 1 mm.

**Figure 8:**
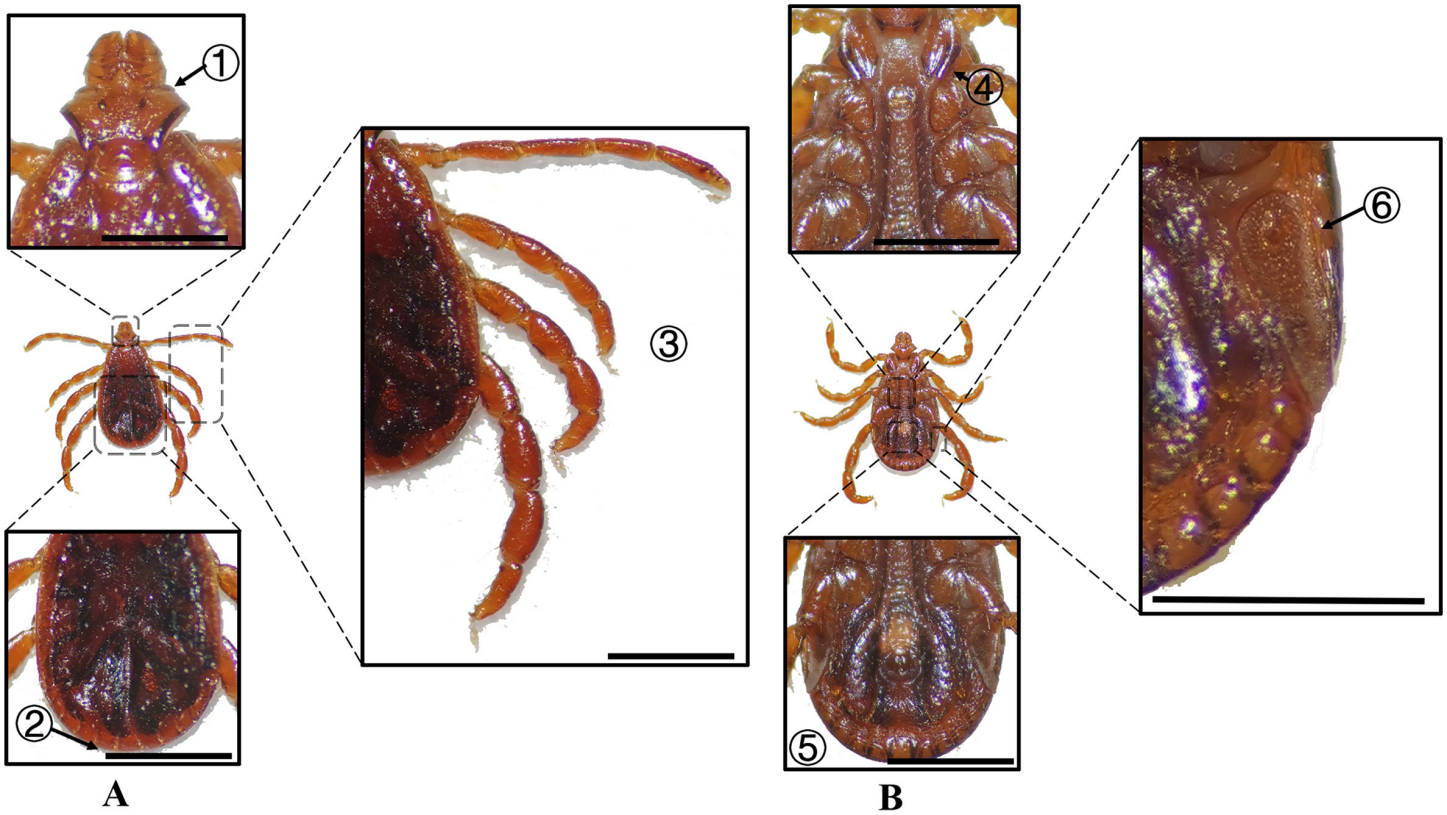
**(A)** Dorsal and **(B)** ventral views of a male *R. turanicus*. Labels: (1) Basis capituli; (2) Festoons; (3) Legs; (4) External and internal spurs of coxae I; (5) Anal sulcus; (6) Spiracular plate. Scale bar = 1 mm.

**Figure 9:**
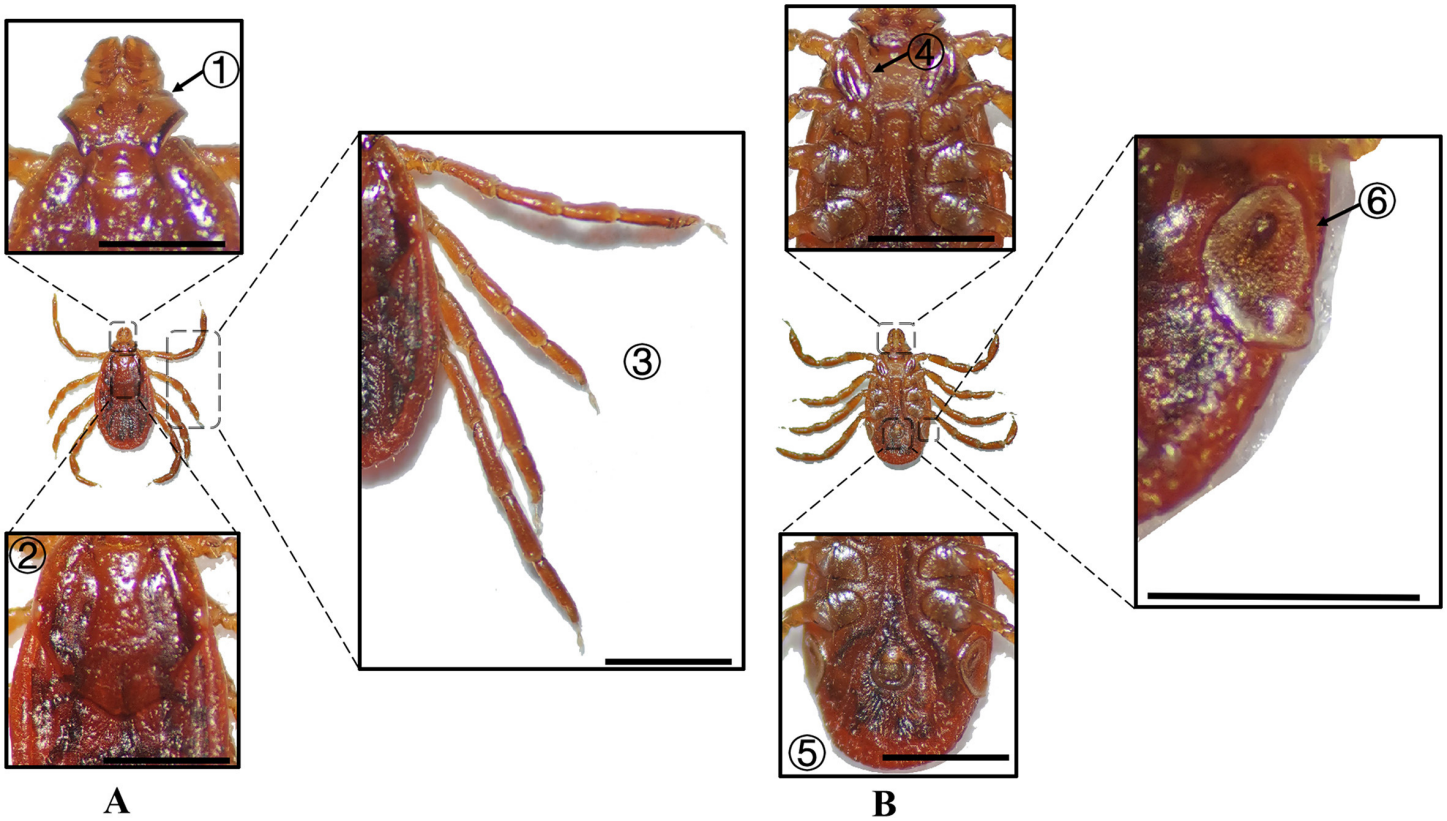
**(A)** Dorsal and **(B)** ventral views of a female *R. turanicus*. Labels: (1) Basis capituli; (2) Scutum; (3) Legs; (4) External and internal spurs of coxae I; (5) Anal sulcus; (6) Spiracular plate. Scale bar = 1 mm.

**Figure 10:**
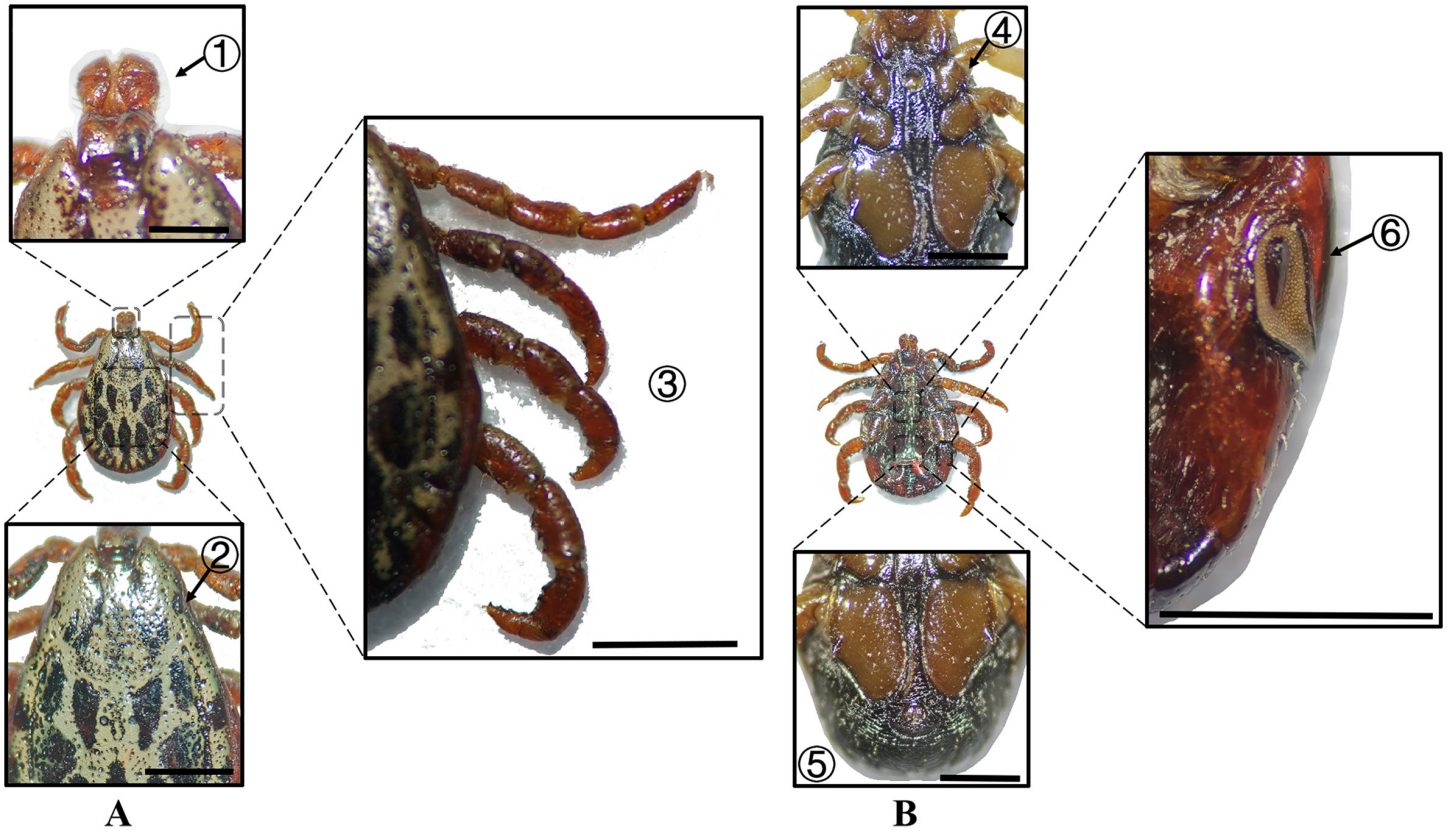
**(A)** Dorsal and **(B)** ventral views of a male *D. marginatus*. Labels: (1) Palps; (2) Eyes; (3) Legs; (4) External and internal spurs of coxae I; (5) Anal sulcus; (6) Spiracular plate. Scale bar = 1 mm.

**Figure 11:**
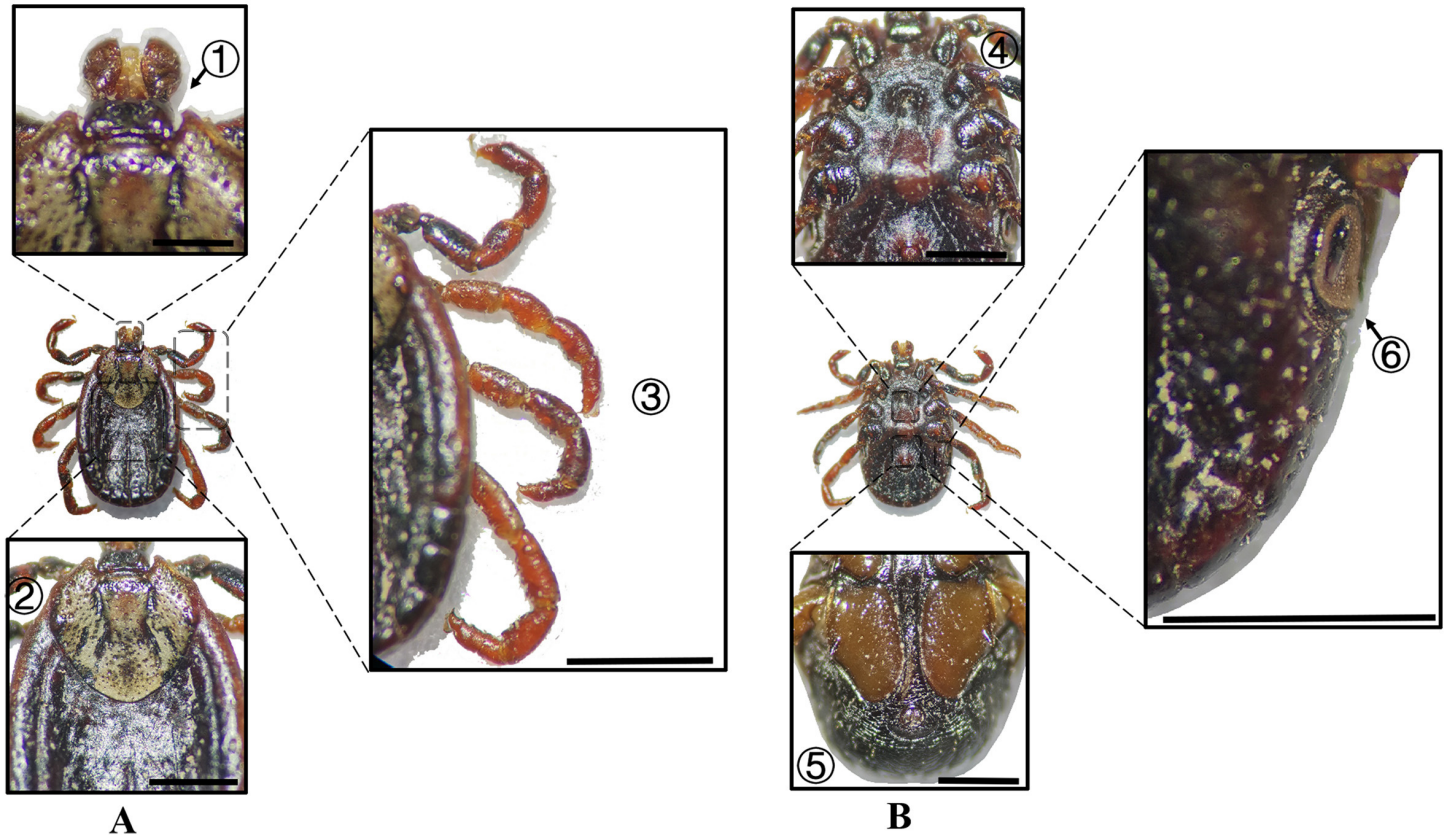
**(A)** Dorsal and **(B)** ventral views of a female *D. marginatus*. Labels: (1) Basis capituli; (2) Scutum; (3) Legs; (4) Genital aperture; (5) Anal sulcus; (6) Spiracular plate. Scale bar = 1 mm.

The capitulum, scutum, and spiracular plates of the collected tick specimens—*Hyalomma anatolicum* (*H. anatolicum*), *Hyalomma asiaticum* (*H. asiaticum*), *Hyalomma dromedarii* (*H. dromedarii), Rhipicephalus turanicus (R. turanicus)*, and *Dermacentor marginatus* (*D. marginatus*)—were examined to determine morphological characteristics and capture high-resolution images, as detailed in Figures 2–11, Table 2 and Supplementary Table S1.

**Table 2:** Morphological characteristics of different tick species.

**Species**	**Sex**	**Figure**	**Dorsum: body size and coloration**	**Basis capituli**	**Legs**	**Abdomen**	**Spiracular plate**
*H. anatolicum*	Male	Figure 2	Small body; elongate-oval reddish-brown dorsum	⋆Sub-trapezoidal with a slightly concave posterior margin	Coxa I with equal external/internal spurs; coxae II–III lack internal spurs; coxa IV with denticle-like spur	Ventral anal plate and accessory adanal plates (broad, short angular projections)	⋆Spatulate with elongated “handle” near the dorsal margin
*H. anatolicum*	Female	Figure 3	Small body size; rhomboid-like yellowish-brown scutum	⋆Sub-trapezoidal with a straight posterior margin; oval porose areas (interporose distance ≈ shorter diameter)	Coxa I with equal spurs; coxae II-IV lack internal spurs	–	Sub-rectangular with right-angled dorsal projection
*H. asiaticum*	Male	Figure 4	Medium-sized; pyriform reddish-brown dorsum	⋆Sub-trapezoidal with a deeply concave posterior margin	Robust elongated legs; coxa I with curved external spur ( ≤ internal spur); coxae II–IV small diminishing spurs	Ventral anal plate and accessory adanal plates (broad triangular projections)	⋆Flask-shaped with narrow elongated “neck” near the dorsal margin
*H. asiaticum*	Female	Figure 5	Medium-sized; dark brown rhomboid scutum (merged cervical and lateral grooves)	⋆Sub-trapezoidal with a straight posterior margin; oval porose areas (interporose distance < shorter diameter)	Robust elongated legs; coxa I with curved external spur ( ≤ internal spur); coxae II–IV small diminishing spurs	–	Elongated oval with right-angled dorsal projection
*H. dromedarii*	Male	Figure 6	⋆Large-sized; oval dark brown dorsum with coarse punctations. Median pale-yellow festoon	Sub-trapezoidal with a shallowly concave posterior margin	Robust legs (IV stoutest); coxa I with equal spurs; coxae II–IV small diminishing spurs	⋆Ventral anal plate > accessory plates; inverted triangular genital apron	Spatulate
*H. dromedarii*	Female	Figure 7	Large-sized; broad dark brown scutum	Broad and short with a convex lateral margin and a straight posterior margin; oval porose areas (interporose distance of ≤ shorter diameter)	–	⋆Genital apron is an inverted triangular	Elongated oval with short dorsal projection and chitinous thickening
*R. turanicus*	Male	Figure 8	Small body; melon-seed-shaped russet-brown dorsum	⋆Hexagonal with prominent basal angles	Moderately stout legs; coxa I with equal external/internal spurs; coxae II–IV small denticle-like spurs; leg IV longest and thickest	Absence of ventral anal plate; elongated-triangular adanal plates; narrow accessory adanal plates	Spatulate with straight broad “handle”, near the dorsal margin
*R. turanicus*	Female	Figure 9	Small body size; elongated-ovoid russet-brown dorsum; rhomboid russet-brown scutum	Hexagonal with prominent basal angles	Coxa I external spur < internal spur; coxae II–IV small denticle-like spurs	⋆Caudal appendage is broad.	Comma-shaped with short weakly developed dorsal projection
*D. marginatus*	Male	Figure 10	⋆Medium-sized; pyriform dorsum; discontinuous porcelaneous enamel patches on lateral scutum	Sub-rectangular with a shallowly concave posterior margin	Robust legs; coxa I external spur < internal spur; coxae II–IV distinct short spurs; coxa IV markedly enlarged	⋆Ventral abdomen lacks chitinous plates	Comma-shaped (large) with short weakly developed dorsal projection
*D. marginatus*	Female	Figure 11	⋆Medium-sized; ovoid dorsum; cordate scutum with porcelaneous enamel patches and scapular apices	Sub-rectangular, twice as wide as long	Stout legs; coxa I external spur < internal spur; coxae II–IV narrow denticle-like spurs; broad discoid callosity at coxal-trochanter	–	Comma-shaped (large) with short weakly developed dorsal projection

⋆ = species-specific diagnostic characteristics.

### 3.2 Phylogenetic analysis results

PCR amplification targeting the mitochondrial 16S rDNA and COI genes, along with the nuclear ITS2 region, was conducted using whole-genome DNA extracted from the ticks. Agarose gel electrophoresis revealed distinct bands of approximately 461 bp (16S rDNA), 709 bp (COI), and 821 bp (ITS2), corresponding to the expected fragment sizes (Supplementary Figures S1–S3 and Supplementary Table S2).

**Figure 12:**
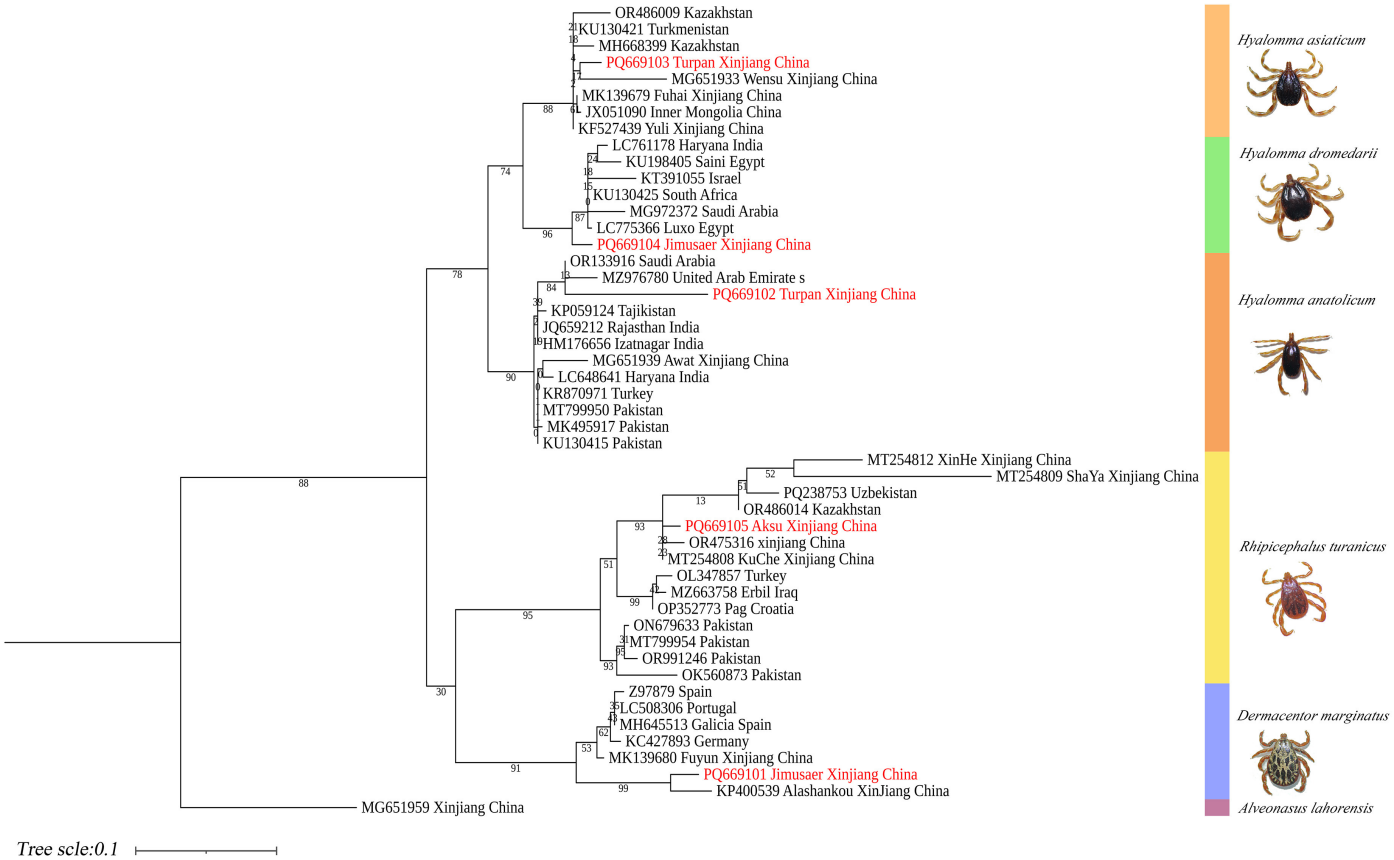
Phylogenetic tree of 16S rDNA genes from five tick species: *H. anatolicum* (PQ669102), *H. asiaticum* (PQ669103), *H. dromedarii* (PQ669104), *R. turanicus* (PQ669105), and *D. marginatus* (PQ669101). *Alveonasus lahorensis* (MG651959) was used as the outgroup. The tree was constructed using the maximum likelihood method under the Kimura 2-parameter + G + I model with 1,000 bootstrap replicates.

Phylogenetic analysis based on the 16S rDNA gene showed that *H. anatolicum* (PQ669102) from Turpan clustered with sequences from the United Arab Emirates; *H. asiaticum* (PQ669103) from Turpan was closely related to populations in Wensu, Xinjiang; *H. dromedarii* (PQ669104) from Jimsar grouped with sequences from Saudi Arabia; *R. turanicus* (PQ669105) from Aksu aligned with Kuqa, Xinjiang; and *D. marginatus* (PQ669101) from Jimsar clustered with sequences from Alashankou, Xinjiang (Figure 12).

**Figure 13:**
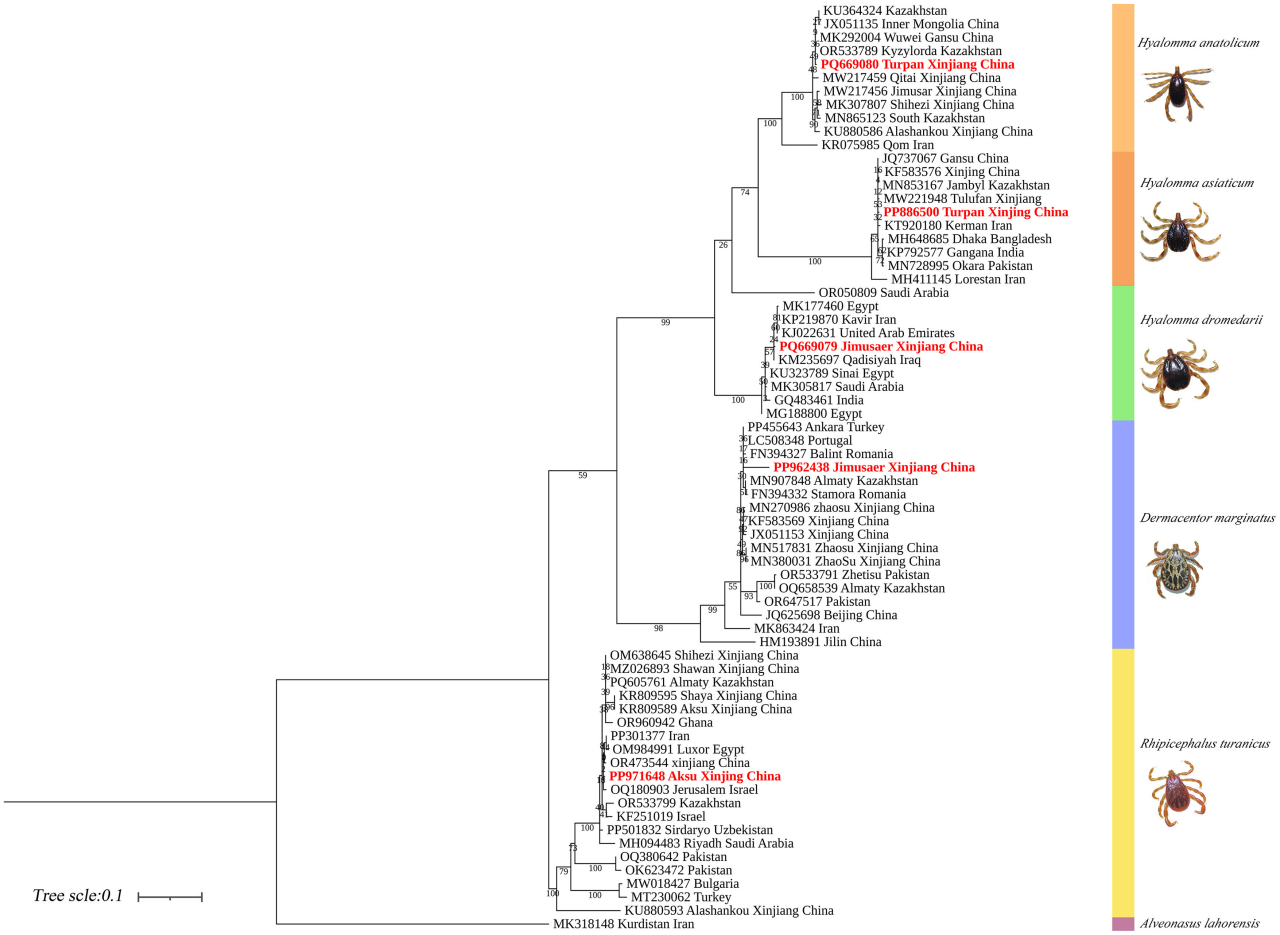
Phylogenetic tree of the COI gene for five tick species: *H. anatolicum* (PP886500), *H. asiaticum* (PQ669080), *H. dromedarii* (PQ669079), *R. turanicus* (PP971648), and *D. marginatus* (PP962438). *Alveonasus lahorensis* (MK318148) was used as the outgroup. The tree was constructed using the maximum likelihood method based on the Tamura-Nei + G + I model with 1,000 bootstrap replicates.

COI-based phylogenetic analysis revealed that *H. anatolicum* (PP886500) from Turpan clustered with specimens from the Zhanbyl Region, Kazakhstan; *H. asiaticum* (PQ669080) aligned with sequences from Kostanay City, Kazakhstan; *H. dromedarii* (PQ669079) grouped with sequences from the United Arab Emirates; *R. turanicus* (PP971648) aligned with samples from Luxor, Egypt; and *D. marginatus* (PP962438) clustered closely with specimens from Almaty, Kazakhstan (Figure 13).

**Figure 14:**
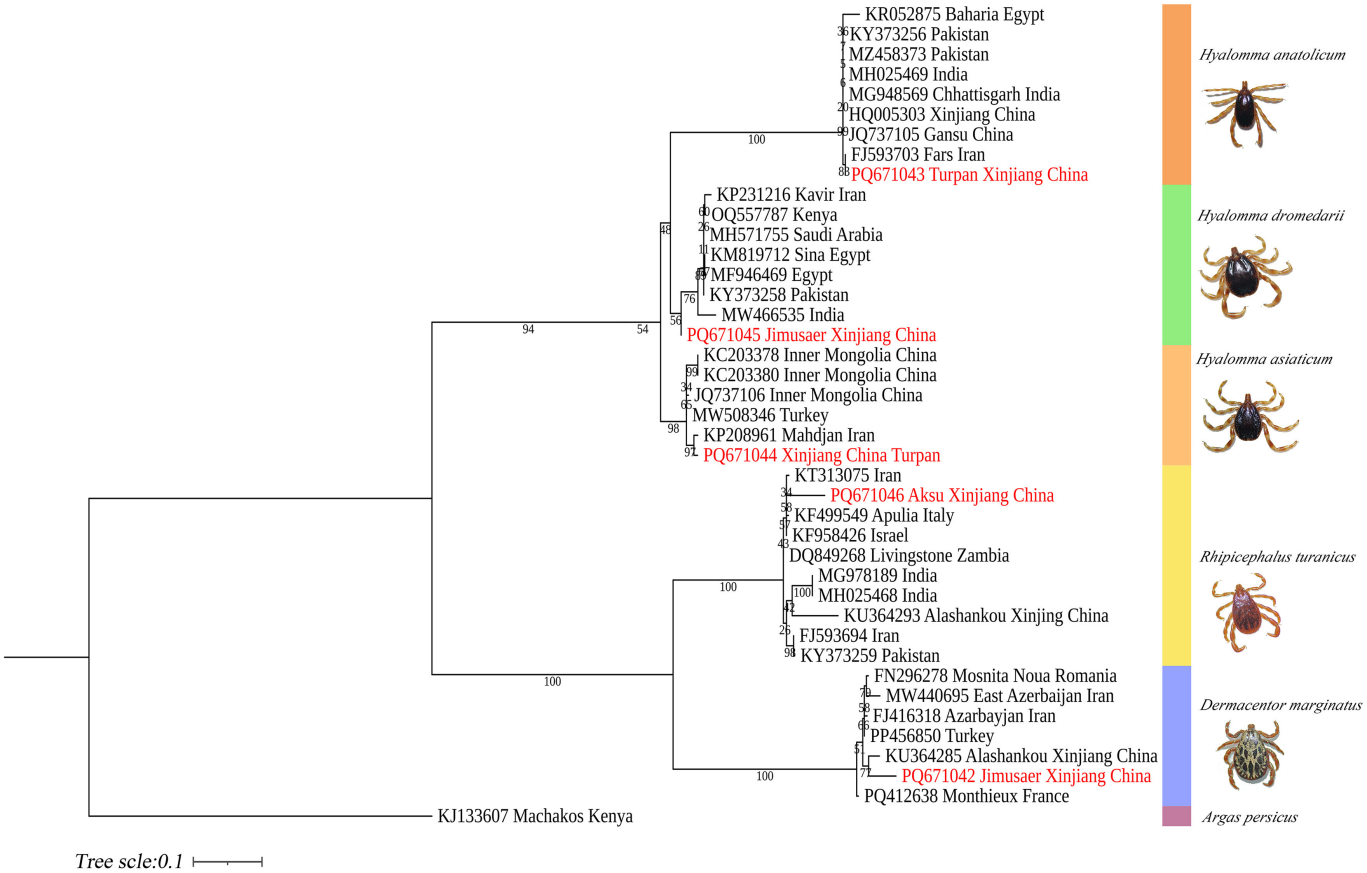
Phylogenetic tree of the ITS2 gene from five tick species: *H. anatolicum* (PQ671043), *H. asiaticum* (PQ671044), *H. dromedarii* (PQ671045), *R. turanicus* (PQ671046), and *D. marginatus* (PQ671042). *Argas persicus* (KJ133607) was used as the outgroup. The tree was constructed using the maximum likelihood method under the Tamura 3-parameter + G model with 1,000 bootstrap replicates.

ITS2-based phylogenetic reconstruction demonstrated that *H. anatolicum* (PQ671043) from Turpan clustered with sequences from Iran; *H. asiaticum* (PQ671044) showed high genetic similarity to populations in Kerman Province, Iran; *H. dromedarii* (PQ671045) grouped with Iranian sequences; *R. turanicus* (PQ671046) aligned with other Iranian samples; and *D. marginatus* (PQ671042) from Jimsar exhibited strong homology with specimens from Alashankou, Xinjiang (Figure 14).

## 4 Discussion

The Xinjiang Uygur Autonomous Region is located in northwest China, bordering the Eurasian continental core with over 5,600 kilometers of international boundaries shared with Russia, Kazakhstan, Kyrgyzstan, Tajikistan, Pakistan, Mongolia, India, and Afghanistan. Xinjiang encompasses extensive natural grasslands and supports a large livestock population. In recent years, its strategic geographic location and the rapid advancement of the Belt and Road Initiative have accelerated cross-border livestock movement, promoting the dissemination of ticks and increasing the risk of tick-borne pathogen transmission (9). Rapid shifts in tick population distributions, coupled with subtle interspecific morphological differences and a lack of high-resolution reference images, have substantially hindered morphological identification—particularly for congeneric species and for researchers without specialized taxonomic training.

In Xinjiang, *H. anatolicum* primarily transmits *T. annulata, Babesia* spp., and *Anaplasma bovis*. Meanwhile (30, 31), *H. asiaticum* serves as a vector for *Rickettsia* spp., *Severe Fever with Thrombocytopenia Syndrome Virus, Babesia caballi*, among others (22). These two species frequently co-occur due to overlapping host preferences and shared ecological niches (32). Morphological differences between *H. anatolicum* and *H. asiaticum* are subtle, particularly in immature or undersized *H. asiaticum* specimens, often leading to misidentification. Key distinguishing features include the posterior margin of the basis capituli in males, which is relatively straight in *H. anatolicum* but deeply concave in *H. asiaticum*. Additionally, the cervical groove in *H. anatolicum* males is shallow and tapers posteriorly, whereas in *H. asiaticum*, it is elongated and prominent, extending to the posterior third of the scutum (13–15). Contrary to previous reports (15), microscopic examination of *H. anatolicum* reveals distinct pale bands near the leg joints and a longitudinal pale stripe along the dorsal margin—features not readily visible under macroscopic observation. In contrast, *H. asiaticum* exhibits clearly visible pale bands at the leg joints under both microscopic and macroscopic conditions. *Hyalomma dromedarii* transmits several pathogens, including *Coxiella burnetii, Borrelia* spp., and *Crimean-Congo hemorrhagic fever virus* (33–35). It is the largest among the collected *Hyalomma* specimens, a trait likely reflecting its adaptation to parasitize camels (26), which possess larger body size, denser fur, and inhabit more extreme environments. Its robust legs, specialized mouthparts, and enlarged digestive system likely enhance its parasitic efficiency and survival. Misidentification with *H. asiaticum* is common; diagnostic features for *H. dromedarii* include: (1) in males, a dark brown ovoid scutum with coarse punctations that are sparsely distributed medially and concentrated laterally; (2) in females, an inverted triangular genital aperture—an important taxonomic marker. *Rhipicephalus turanicus* is a known vector for *Babesia ovis, Babesia caballi*, and other pathogens (6, 36). It exhibits a compact body and is often confused with *R. sanguineus sensu stricto (s.s.)*, particularly in females. Both sexes of *R. turanicus* have short, stout palps, and males present distinct lamellar projections on the ventral side of the basis capituli, with dorsal extensions visible (26, 27, 37). Although broad festoons and caudal processes are typical diagnostic traits in males, the *R. turanicus* specimens collected in this study lacked these features. Nevertheless, they clustered clearly in molecular phylogenetic analyses (28). *Dermacentor marginatus* primarily transmits *Rickettsia* and *Anaplasma* spp., among other pathogens (38, 39). It displays enamel markings characteristic of the Dermacentor genus, facilitating rapid differentiation from other genera (29). However, *D. marginatus* exhibits minimal morphological divergence from its congeners *D. nuttalli* and *D. silvarum*. Therefore, molecular approaches are recommended to improve taxonomic resolution.

In this study, 788 ticks (~70% of the total) were collected from cattle in Turpan City. Among them, *H. anatolicum* exhibited close phylogenetic (9, 24, 32, 40) relationships with populations from Kazakhstan and Iran, likely due to the shared arid, high-temperature temperate continental climate that favors its survival. *H. asiaticum* specimens also showed close genetic affinities with Iranian populations. Despite sharing ecological niches with *H. anatolicum*, its three-host life cycle and longer developmental duration may constrain dispersal, limiting its genomic representation. *H. dromedarii* from Jimsar County clustered phylogenetically with Iranian populations. This may be attributed to historical camel husbandry and trade, facilitating gene flow. *R. turanicus* specimens from Aksu—geographically near Central Asia—exhibited close affinities with Iranian and Egyptian populations. Ancient host migration routes, such as the Silk Road, and convergent ecological adaptations may have maintained genetic connectivity (41). *Dermacentor marginatus* from Jimsar County showed strong phylogenetic similarity with specimens from Alashankou City, Xinjiang. This may result from environmental similarities (e.g., arid semi-desert steppe), seasonal livestock migration, or tick dispersal via migratory birds along the Alashankou corridor (38). Most ticks collected in this study were from bovines and ovines in the Turpan region (70% of total), while samples from camelids (*Junggar Bactrian camels*) and canines (*Chinese rural dogs*) were limited. This sampling imbalance may restrict interpretations of host-specific transmission dynamics of tick-borne pathogens.

Molecular identification is unaffected by developmental stage (larva, nymph, adult) or morphological integrity, thus overcoming the limitations of morphology-based taxonomy. By targeting signature genes, interspecies differentiation can be achieved, even among morphologically similar taxa. Three molecular markers offer complementary advantages: The 16S rDNA gene effectively reveals genetic differentiation driven by geographic isolation in population studies, but offers limited resolution for closely related species (17, 20, 42, 43). The COI gene (cytochrome oxidase I): a mitochondrial gene with high copy numbers, enabling efficient PCR amplification even from degraded or low-quantity samples (20). ITS2: a nuclear ribosomal DNA marker subject to lower selective pressure and higher evolutionary rates, resulting in greater interspecific variability than mitochondrial genes, making it ideal for resolving closely related taxa (20, 44). However, overreliance on ITS2 alignments in open-access databases such as NCBI can introduce misidentification due to annotation errors (45). For instance, in this study, *R. turanicus* ITS2 sequences showed 99% similarity with *R. sanguineus* in GenBank, likely stemming from early taxonomic ambiguities. The *R. sanguineus* group has long been taxonomically unstable due to overlapping morphological features with *R. turanicus*, lack of designated type specimens, potential hybridization, and limited diagnostic tools. Consequently, *R. sanguineus* s.s. and *R. turanicus* were frequently misidentified under the umbrella term “*R. sanguineus*” (27, 37). Recent taxonomic revisions now recognize *R. sanguineus s.s*. and *R. turanicus* as distinct species. The term “*R. sanguineus sensu lato* (*s.l*.)” is recommended for ticks morphologically resembling *R. sanguineus* but lacking genetic confirmation or showing divergent genotypes (37). To resolve these challenges, integrative analyses incorporating both mitochondrial and nuclear markers are strongly recommended to improve species delimitation and mitigate database inconsistencies among cryptic taxa (20, 45, 46).

Accurate identification of tick species requires the integration of morphological characterization and molecular diagnostics. By combining analyses of morphological traits with genetic sequence alignment, this dual approach enables precise taxonomic classification and robust phylogenetic inference. This multidimensional strategy addresses the limitations inherent in single-method identifications and generates critical data for elucidating ecological transmission pathways of tick-borne diseases and pathogen evolution.

## 5 Conclusions

The integrative morphological-molecular framework effectively resolves taxonomic ambiguities among five tick species, wherein diagnostic traits—such as capitulum morphology and spiracular plate structure—complement multi-locus phylogenetic analyses based on 16S rDNA, COI, and ITS2 sequences. Genetic affinities observed between tick populations in Xinjiang and lineages from Central Asia and the Middle East suggest that transboundary dispersal is likely facilitated by livestock trade and climate-driven habitat shifts. The limitations of single-gene identification methods and the presence of cryptic species (e.g., *Rhipicephalus turanicus* vs. *R. sanguineus sensu stricto*) underscore the necessity for high-resolution imaging and multi-gene validation to ensure accurate species delimitation. This framework enhances global tick surveillance capacity and informs targeted control strategies in increasingly dynamic ecological landscapes.

## Data Availability

The sequences obtained in this study have been deposited in the NCBI GenBank database under the following accession numbers: 16S rDNA gene (PQ669101-PQ669105), COI gene (PP886500, PQ669080, PQ669079, PP971648, PP962438), and ITS2 gene (PQ671042-PQ671046). All other data generated or analyzed during this study are available in the published article and its Supplementary Information files.
