# Supplementary material for: Integrative morpho-molecular delineation of five medically significant tick species: facilitating precision-based vector surveillance
Source: Front Vet Sci. 2025 Aug 8;12:1623318. doi: 10.3389/fvets.2025.1623318 (PMC12371277; doi:10.3389/fvets.2025.1623318)
Supplement: Supplementary file 1 [file Table_1.pdf]

Note: Supplementary data associated with this article

Supplementary Table S1. High-resolution images of ticks

	Overall	Basis capituli	Upper half	Lower half	Legs /Spiracular plate
Dorsal views of a male *H.anatolicum*	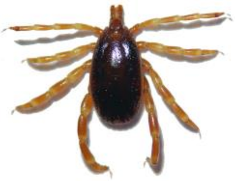	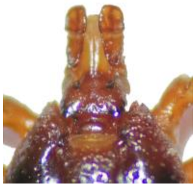	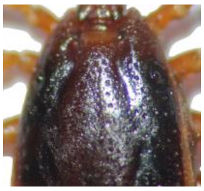	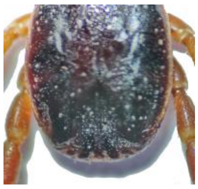	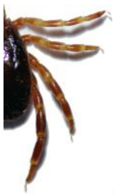
Ventral views of a male *H.anatolicum*	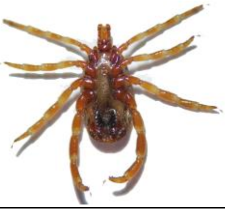	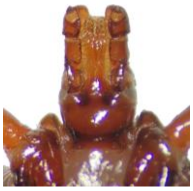	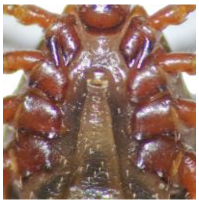	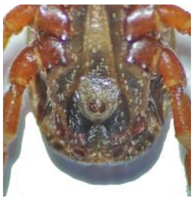	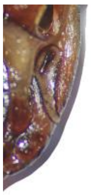
Dorsal views of a female *H.anatolicum*	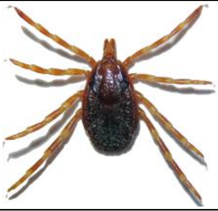	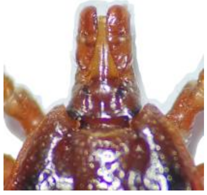	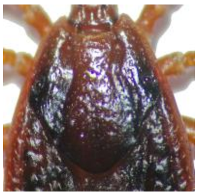	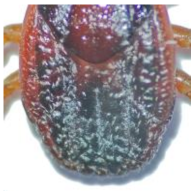	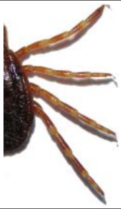
Ventral views of a female *H.anatolicum*	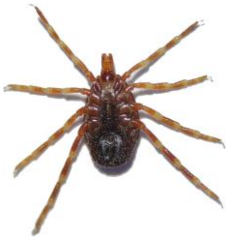	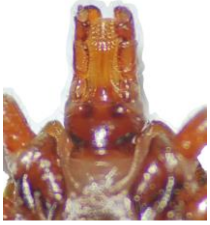	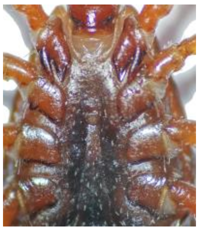	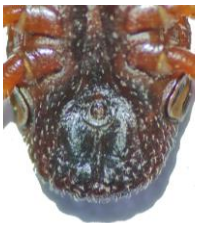	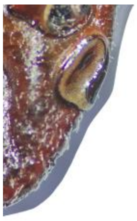
Dorsal views of a male *H.asiaticum*	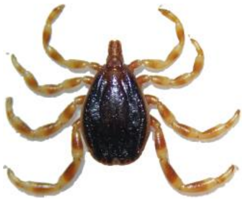	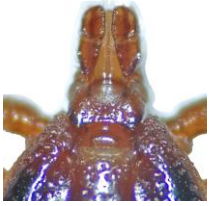	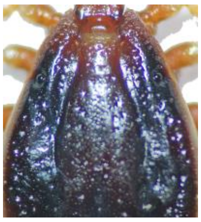	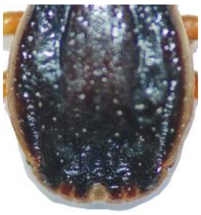	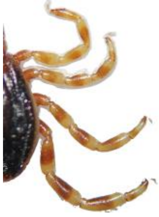
Ventral views of a male *H.asiaticum*	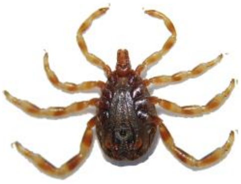	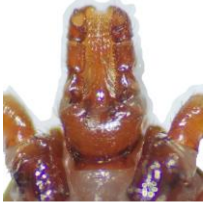	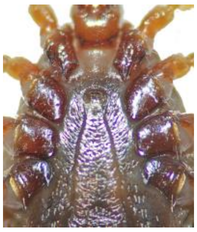	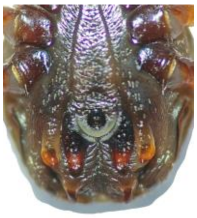	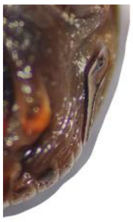
Dorsal views of a female *H.asiaticum*	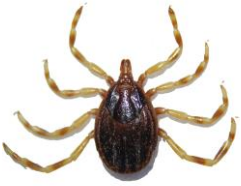	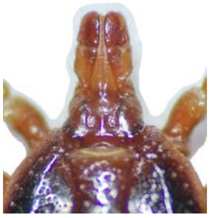	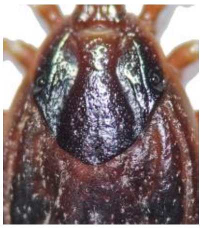	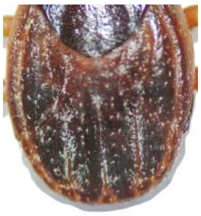	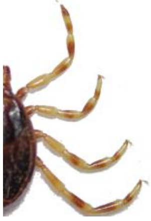

Ventral views of a female *H.asiaticum*	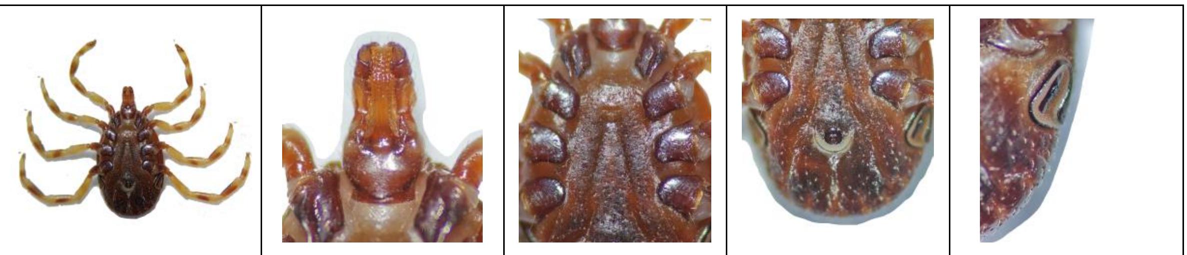
Dorsal views of a male *H.dromedarii*	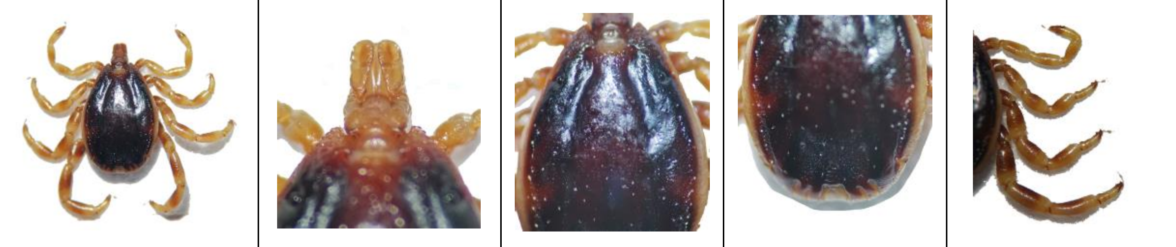
Ventral views of a male *H.dromedarii*	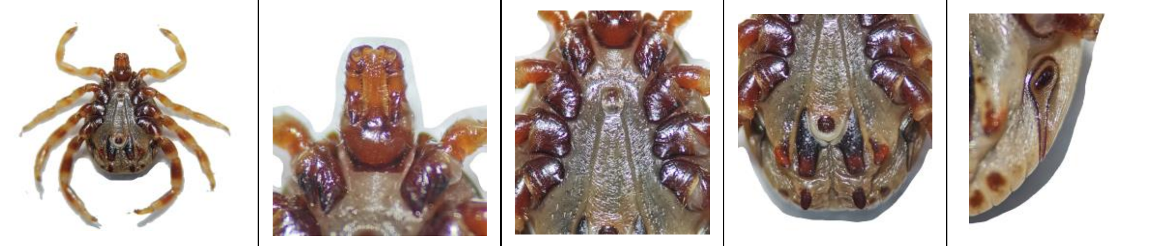
Dorsal views of a female *H.dromedarii*	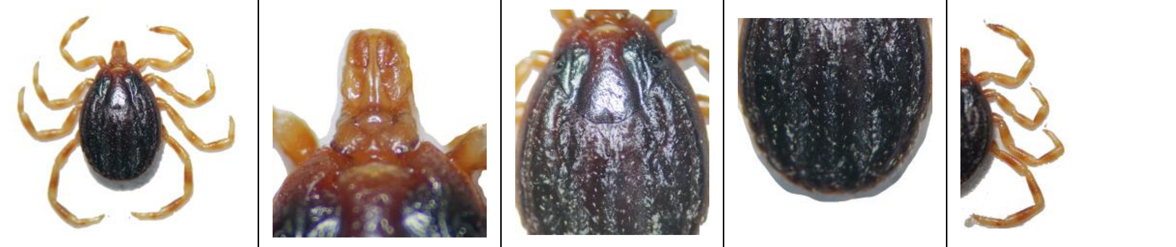
Ventral views of a female *H.dromedarii*	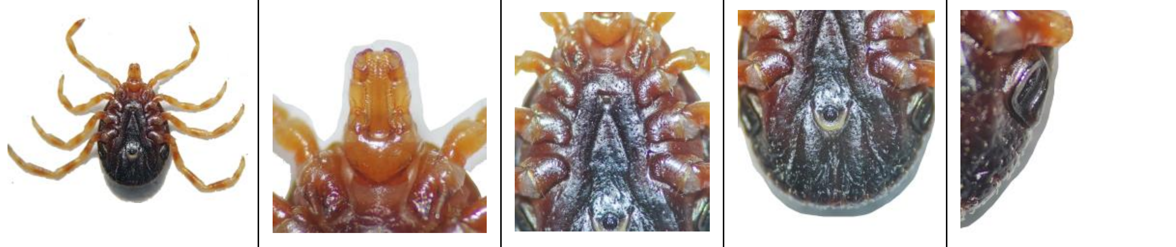
Dorsal views of a male *R.sanguineus*	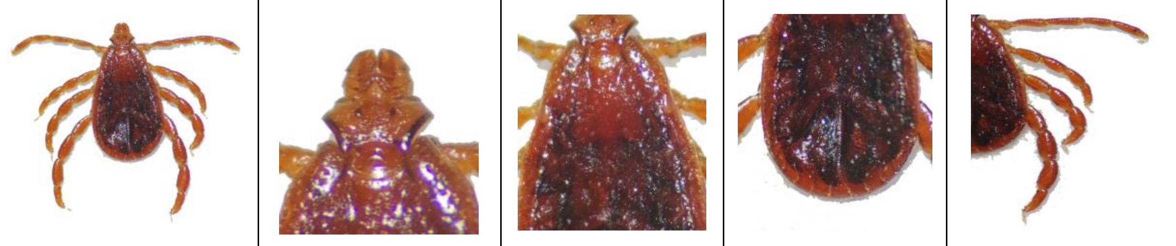
Ventral views of a male *R.sanguineus*	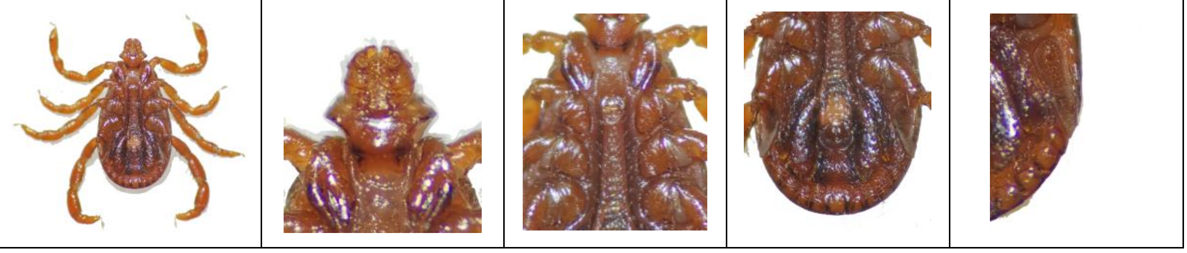

Dorsal views of a female *R.sanguineus*	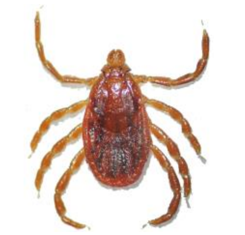	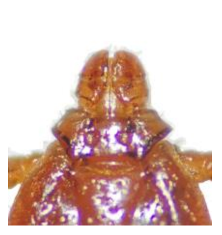	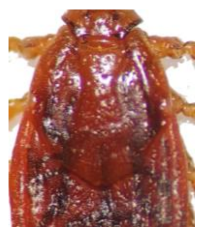	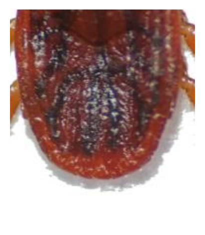	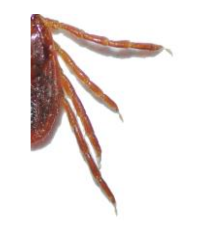
Ventral views of a female *R.sanguineus*	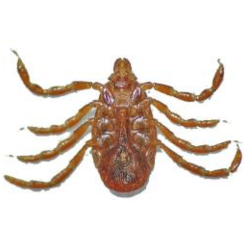	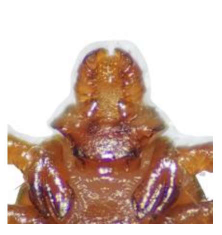	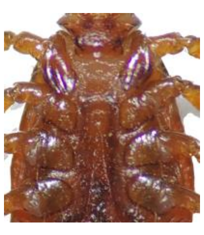	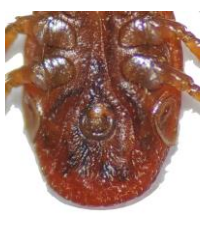	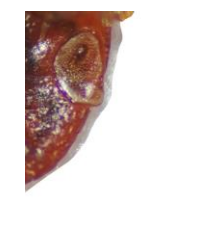
Dorsal views of a male *D.marginatus*	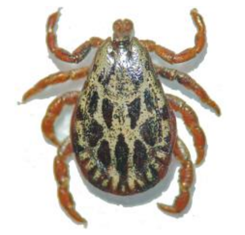	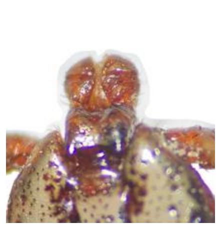	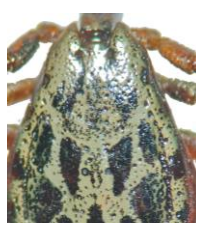	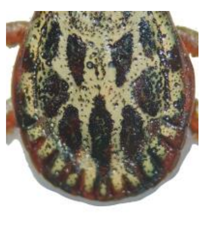	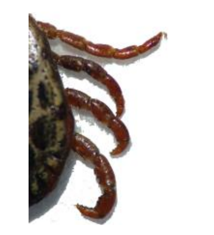
Ventral views of a male *D.marginatus*	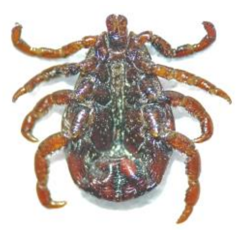	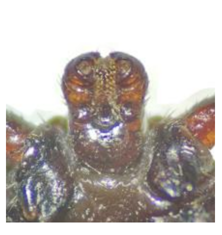	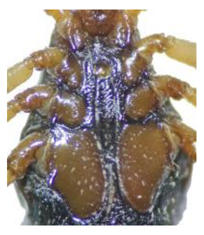	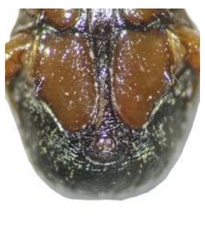	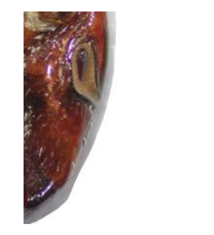
Dorsal.view s of a female *D.marginatus*	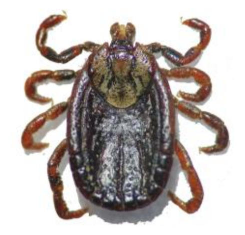	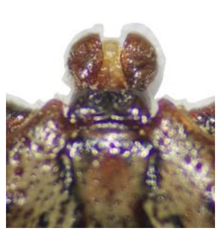	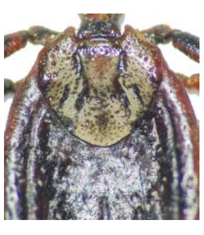	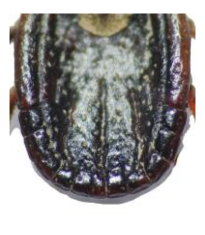	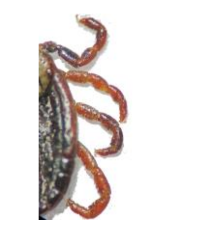
Ventral views of a female *D.marginatus*	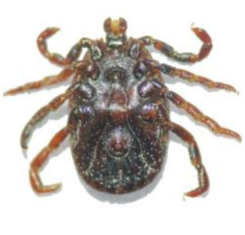	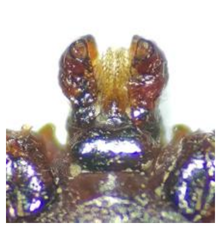	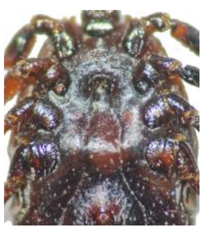	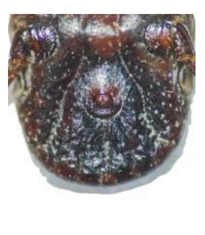	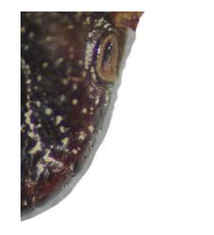
