# Supplementary material for: Integrative morpho-molecular delineation of five medically significant tick species: facilitating precision-based vector surveillance
Source: Front Vet Sci. 2025 Aug 8;12:1623318. doi: 10.3389/fvets.2025.1623318 (PMC12371277; doi:10.3389/fvets.2025.1623318)
Supplement: Supplementary file 2 [file Table_2.docx]

| Supplementary Table S2 PCR validation results | | | | | | | |
| --- | --- | --- | --- | --- | --- | --- | --- |
| Species | Geographical origin | Hosts | 16S rDNA PCR Result | | COI PCR Result | ITS2 PCR Result | |
| *H.anatolicum* | Turpan（6） | *Simmental cattle*,  *Altay sheep* | 100% | 100% | | | 100% |
| *H.asiaticum* | Turpan（6） | *Simmental cattle*,  *Altay sheep* | 100% | 100% | | | 100% |
| *H.dromedarii* | Jimsar（3） | *Bactrian camel* | 100% | 100% | | | 100% |
| *R. turanicus* | Aksu（3） | *Altay sheep* | 100% | 100% | | | 100% |
|  | Urumqi（3） | *Chinese rural dog* | 100% | 100% | | | 100% |
| *D.marginatus* | Jimsar（3） | *Altay sheep* | 100% | 100% | | | 100% |
