# Supplementary figures and images for: Integrative morpho-molecular delineation of five medically significant tick species: facilitating precision-based vector surveillance
Source: Front Vet Sci. 2025 Aug 8;12:1623318. doi: 10.3389/fvets.2025.1623318 (PMC12371277; doi:10.3389/fvets.2025.1623318)

Note: Supplementary data associated with this article

Supplementary Figure

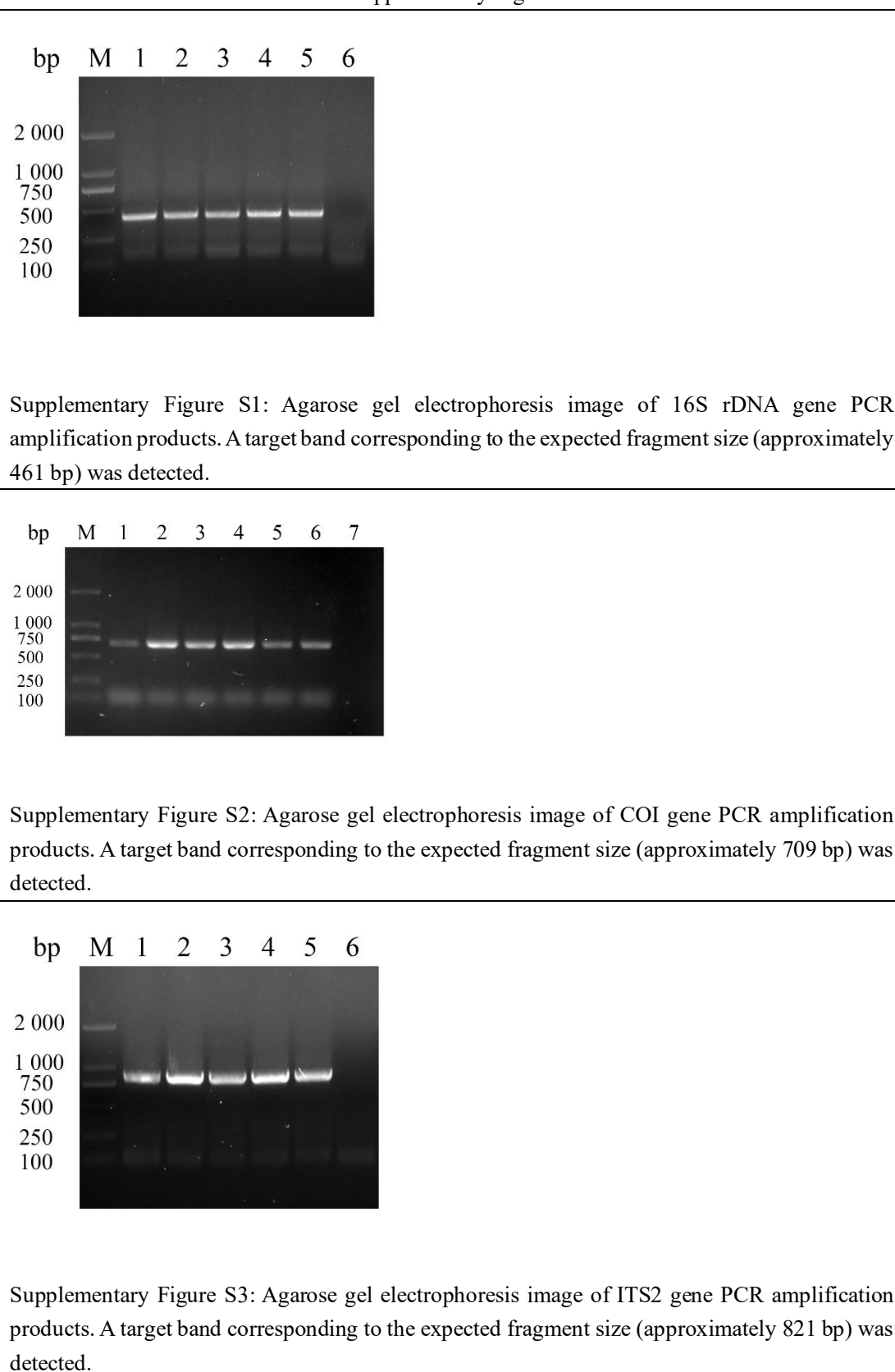

Supplement: Supplementary file 3 [file Image_1.pdf]
